# Impact of benzannulation on ESIPT in 2-(2′-hydroxyphenyl)-oxazoles: a unified perspective in terms of excited-state aromaticity and intramolecular charge transfer†

**DOI:** 10.1039/d0ra05802e

**Published:** 2020-10-23

**Authors:** Leandro D. Mena, D. M. A. Vera, María T. Baumgartner

**Affiliations:** INFIQC, Departamento de Química Orgánica, Facultad de Ciencias Químicas, Universidad Nacional de Córdoba, Ciudad Universitaria Córdoba X5000HUA Argentina lmena@fcq.unc.edu.ar tere@fcq.unc.edu.ar; QUIAMM-INBIOTEC-Departamento de Química, Facultad de Ciencias Exactas y Naturales, Universidad Nacional de Mar del Plata Mar del Plata Argentina

## Abstract

Hydroxyphenyl-azoles are among the most popular ESIPT (Excited State Intramolecular Proton Transfer) scaffolds and as such, they have been thoroughly studied. Nevertheless, some aspects regarding the interplay between the emissive properties of these fluorophores and the size of their π-conjugated framework remain controversial. Previous studies have demonstrated that benzannulation of 2′-hydroxyphenyl-oxazole at the phenol group of the molecule can lead to either red- or blue-shifted fluorescence emission, depending on the site where it occurs. In this report, benzannulation at the heterocyclic unit (the oxazole site) is analysed in order to get the whole picture. The extension of π-conjugation does not significantly affect the ESIPT emission wavelength, but it leads instead to higher energy barriers for proton transfer in the first excited singlet state, as a consequence of dramatic changes in the charge transfer character of excitation caused by successive benzannulation. Theoretical calculations revealed an interesting connection between intramolecular charge transfer and excited-state aromaticity in the S_1_ state. The theoretical approach presented herein allows the behaviour of hydroxyphenyl-oxazoles in the excited state to be rationalized and, more generally, a deeper understanding of the factors governing the ESIPT process to be obtained, a crucial point in the design of new and efficient fluorophores.

## Abstract 2

Benzannulation of a typical fluorophore reveals the interplay between ESIPT, excited-state aromaticity and intramolecular charge transfer.

## Graphical abstract

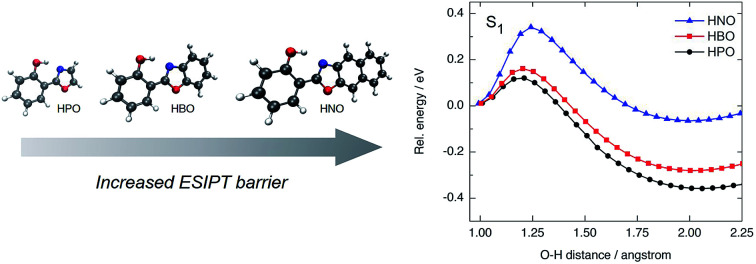

## Introduction

The design of organic fluorophores with a π-conjugated system has become a fundamental part in the development of functional materials such as photovoltaic devices^1^ and organic light-emitting diodes (OLEDs).^2^ In this context, it is highly desirable to obtain red-shifted emission with large Stokes shifts, in order to minimize self-absorption effects and to obtain low signal-to-noise ratios for bioimaging applications.^3^ Emitters based on ESIPT (Excited State Intramolecular Proton Transfer) have emerged as an interesting class of fluorophores since their emission usually occurs with anomalously high Stokes shifts (6000–12 000 cm^−1^). As the ESIPT process is essentially an excited-state tautomerization, the keto tautomer formed upon photoexcitation (K*) is structurally different from the starting enol form (E), which causes the former to exhibit red-shifted fluorescence emission. In some cases, it is also possible to obtain dual fluorescence from both enol and keto forms, a unique feature that has been applied to the development of white light-emitting materials.^4^ Additionally, ESIPT compounds present intense solid-state emission, a significant advantage over common fluorophores which usually undergo aggregation-induced fluorescence quenching in films or crystals.^5^

It is well known that, together with the introduction of electron donor and acceptor groups into the structure of a chromophore,^6^ the modification of π-conjugation is the most common way to tune the absorption energy. In general, extending conjugation results in bathochromic shifts in absorption. Although the same is true for fluorescence in many systems,^7^ this rule of thumb does not always apply for molecules that undergo significant structural changes in excited state. A particular example of this is the case of ESIPT fluorophores. Despite the excited state behaviour of this kind of molecules has been extensively studied, the influence of the conjugation extension on fluorescence emission remained controversial during the last decades.

**Scheme 1:**
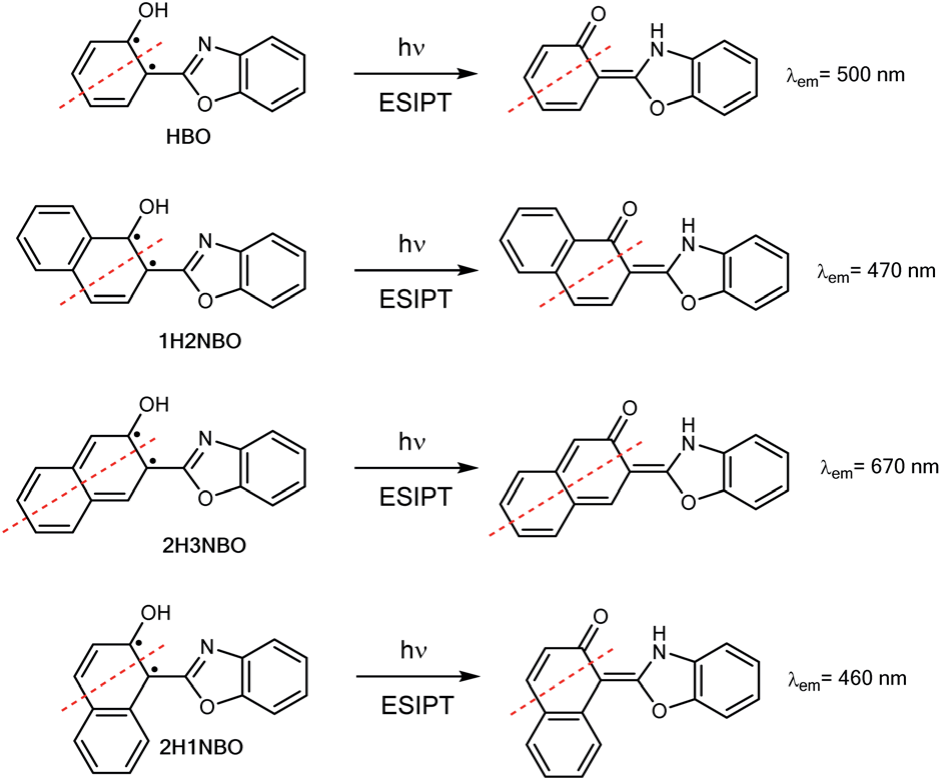
ESIPT in HBO and its benzannulated derivatives 2-(1′-hydroxy-2′-naphthyl)-benzoxazole 1H2NBO, 2-(2′-hydroxy-3-naphthyl)-benzoxazole 2H3NBO and 2-(2′-hydroxy-1-naphthyl)-benzoxazole 2H1NBO. Red broken lines indicate the nodal plane orthogonal to the molecular plane; dots indicate lone π electrons.

Hydroxyphenyl-azoles represent an archetypical family of ESIPT fluorophores, among which 2-(2′-hydroxyphenyl)-benzoxazole (HBO, Scheme 1) is maybe one of the most studied.^8^ In 1999, Nagaoka *et al.* were pioneers in suggesting that ESIPT emission of HBO depends on the size of the π-system of the phenol part of the molecule.^9^ The authors interpreted the experimental results based on the “nodal plane model”, a qualitative method according to which the ESIPT behaviour can be rationalised by considering the nodal plane of the wavefunction in excited state.^10^ As a proof-of-concept, they studied two hydroxynaphthyl derivatives of HBO bearing 1-naphthol and 2-naphthol units, 1H2NBO and 2H3NBO (Scheme 1), and demonstrated that the emissive properties of both dyes were actually very different: the emission maxima of 1H2NBO is at 470 nm in hexane, whereas 2H3NBO shows ESIPT emission at 670 nm. In their report, the authors suggested that an effective red shift in emission can take place only if the extension of conjugation occurs along the nodal plane of the wavefunction in excited state, since in that way the keto tautomer becomes specially stabilized.

Ten years after Nagaoka's seminal work, the Arai group reported the ESIPT behaviour of 2-(2′-hydroxynaphthalenyl)-benzoxazole (2H1NBO, Scheme 1), another benzannulated derivative of HBO displaying unusually small Stokes shift.^11^ The authors then expanded the investigation toward other naphthalene-fused 2-(2′-hydroxyaryl)benzazoles, finding that ESIPT emission of these dyes was blue-shifted compared to the model compound HBO, but without proposing a possible explanation for this effect.^12^ Almost another ten years later, this unconventional behaviour has been revisited by different authors^13,14^ under the light of the Baird's rule.^15^ This rule, according to which [4*n* + 2] π-aromatic annulenes become antiaromatic in the ππ* S_1_ or T_1_ state, has been successfully applied to rationalize the ESIPT emission profile of different benzannulated HBO derivatives by connecting the relative stability of the tautomers to their aromatic (antiaromatic) character in the ground (excited) state.

To the best of our knowledge, these antecedents only deal with the benzannulation of the phenol unit of HBO, that is, the donor part of the molecule. Nevertheless, the effects that benzannulation at the acceptor unit causes on ESIPT still remain unknown. Considering that the study of both the donor and the acceptor moieties of an ESIPT molecule is essential to get a complete understanding of the phenomenon, we present herein a thorough analysis aimed to clarify the impact that an increase in the π-conjugation of the oxazole unit in HBO has on the ESIPT process. Experimental results demonstrate that benzannulation does not necessarily lead to a significant red-shifted keto emission. In addition, DFT and TDDFT calculations enabled us to provide a reasonable explanation for the observed behaviour and to get deeper insights into the ESIPT reactivity of the studied system. Finally, a global comparison with previous results is proposed, revisiting the behaviour of the ring-fused phenol derivatives under the light of the Baird's and Clar's rules.

## Results and discussion

**Scheme 2:**
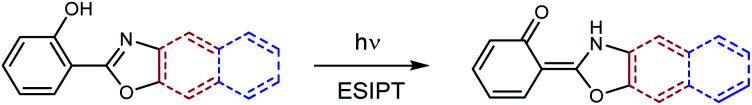
ESIPT reaction in HPO and its benzannulated derivatives, HBO (red) and HNO (blue).

To begin with the study, we propose a comparison between the ESIPT reaction in three azoles with heterocyclic systems of different length: 2-(2′-hydroxyphenyl)-oxazole (HPO), 2-(2′-hydroxyphenyl)-benzoxazole (HBO) and 2-(2′-hydroxyphenyl)-naphthoxazole (HNO) (Scheme 2). For this purpose, we have synthesized and characterized HNO, since the data available in literature about him was scarce. Throughout this text, the experimental data measured for HNO is contrasted to the data extracted from literature about HBO and HPO. Computational results presented herein regarding the three compounds were entirely calculated by us.

### Spectroscopic properties

**Fig. 1:**
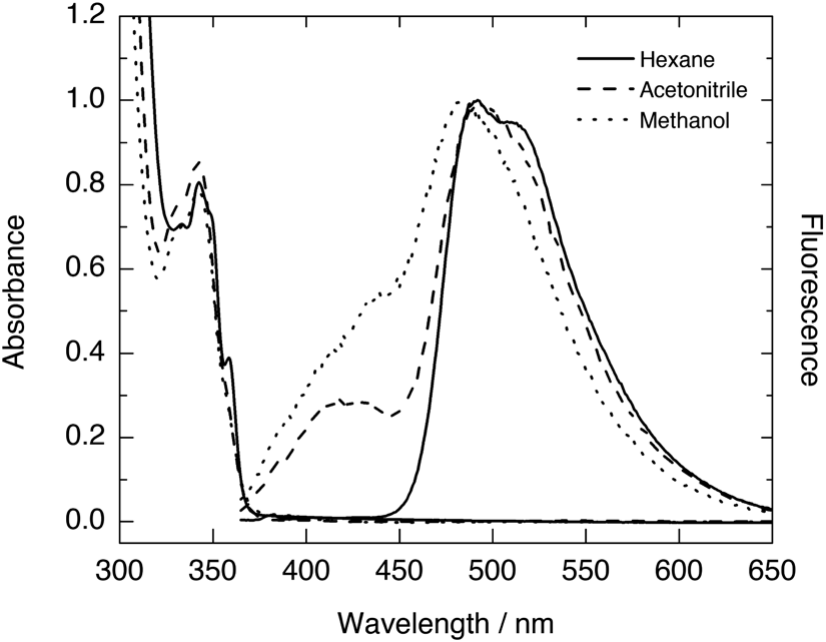
UV-vis absorption and normalised fluorescence spectra of HNO in various solvents.

The UV-vis absorption spectra of HPO exhibit an S_0_ → S_1_ (ππ*) transition with a maximum around 306–310 nm in different solvents according to literature,^16^ whereas the maximum absorption reported for the same transition in HBO is located around 330–334 nm (Table 1).^8^ This maximum is slightly red shifted (at 342–343 nm) in the case of HNO (Fig. 1). Clearly, the extension of conjugation leads to bathochromically shifted absorption, irrespective of the solvent employed.

**Table 1:** Experimental UV-vis absorption and emission maxima for each compound in nm^h^

Dye	*λ*_max_ absorption (enol form)	*λ*_max_ emission (enol and keto forms)	
		*λ*_enol_	*λ*_keto_
HPO*	306^a^, 306^b^, 309^c^	340^g^, 345^a^, 345^b^	480^g^, 460^a^, 465^b^
HBO*	330^d^, 334^c^, 333^e^	362^c^, 365^d^, 351^b^	500^c^, 474^d^, 485^b^
HNO	343^f^, 342^e^, 342^d^	415^b^, 430^d^	493^c^, 483^d^, 493^b^

a Ethanol.

b Acetonitrile.

c Hexane.

d Methanol.

e Dichloromethane.

f Cyclohexane.

g Methylcyclohexane.

h *Data extracted from ref. 16 for HPO and from ref. 8*d* and *i* for HBO.

The fluorescence emission spectra of HPO and HBO available in literature present dominant K* emission in non-polar environments, and dual emission from E* and K* forms in polar solvents such as methanol or acetonitrile (Table 1). The steady-state fluorescence spectra of HNO at room temperature also show single K* emission in *n*-hexane and dual emission in methanol and acetonitrile (Fig. 1). The somewhat structured K* band in *n*-hexane, with a shoulder at 510 nm, is characteristic of the naphthoxazole moiety.^17^ This emission band becomes structureless but does not undergo significant spectral shift in going from hydrocarbon to more polar solvents (Table 1). Unlike this almost solvent polarity-independent K* fluorescence, E* emission exhibits a perceptible change from 415 nm in acetonitrile to 430 nm in methanol (Fig. 1). This behaviour agrees with an E* form with a strongly separated electronic charge in S_1_ and with a K* form in which polarization is compensated with the proton translocation.^18^ In HBO and HPO this effect is logically less pronounced, as they undergo a smaller change in polarization of the enol tautomer upon excitation. Notably, the K* emission wavelength in hydrocarbon solvents slightly changes when going from HPO (480 nm in methylcyclohexane^16^) to HBO (500 nm in *n*-hexane^8*d*^) to HNO (493 nm in *n*-hexane). In contrast to the observed by Nagaoka^10^ and Ijima^12^ for naphtha- and anthra-derivatives of HBO, our results suggest that benzannulation at the oxazole moiety exerts little impact on proton-transfer emission.

### Vertical transitions energies

**Fig. 2:**
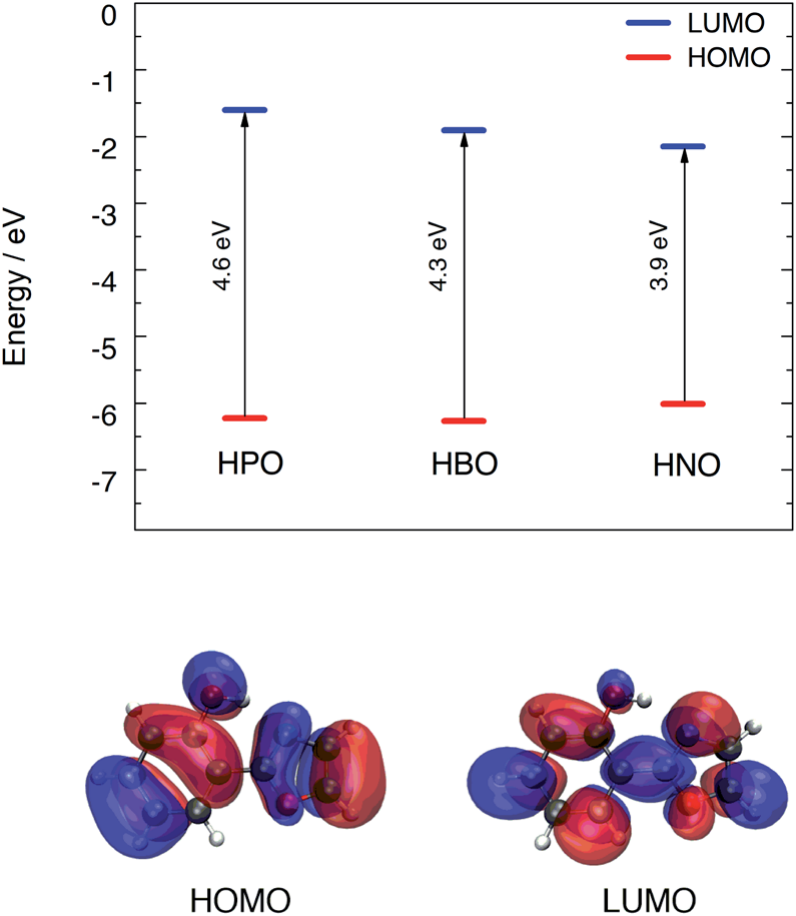
Top: HOMO–LUMO energy levels for the three compounds calculated with B3LYP/6-31+G(d) in acetonitrile. Bottom: HOMO and LUMO distribution for HPO.

Computational modeling of the vertical S_0_ → S_1_ transition using time-dependent density functional theory (TDDFT) for HPO, HBO and HNO at B3LYP/6-31+G(d) level in acetonitrile predicted energies of 4.02 eV (308 nm), 3.77 eV (329 nm) and 3.41 eV (363 nm), respectively. Despite the fact that vertical energies are used in most of the benchmarks to compare with the experimental *λ*_max_ and, in addition, some authors recommend their use,^19,20^ it should be noted that these values are vibrationless difference between S_0_ and S_1_.^21^ This implies an extra approximation that could introduce an additional error added to the errors inherent to the functional and the solvent model. However, these values are useful to rationalize the changes between different compounds and different solvents. According to DFT calculation, the HOMO → LUMO transition is the dominant contributor to the first singlet excited state of the three compounds (see ESI† for MOs depiction). The computed HOMO–LUMO gap energies are close to the excitation energies of the three molecules in acetonitrile. Elongation of π-system decreases the HOMO–LUMO gap mainly due to LUMO stabilization (Fig. 2), which is related to the red shift in absorption observed in going from HPO to HNO.

For fluorescence, the vertical S_1_ → S_0_ energies at the S_1_ geometries were calculated for the enol and keto forms of the three compounds in acetonitrile employing the linear response model of PCM (LR-PCM). B3LYP/6-31+G(d) predicts energies that are in close agreement with experimental data, even without accounting for vibrational effects (Table 2). Other popular functionals lead to considerable deviations, as it is shown in Table 3 for HNO. In general, all the tested DFT functionals predict blue-shifted emissions if compared with B3LYP, for either enol or keto isomers. Hybrid functionals B3LYP and PBE0 give the values closest to the experimental energies, whereas the meta-hybrid GGA functional M06-2X, the range-separated functional CAM-B3LYP and the dispersion-corrected functional ωB97XD overestimate the emission energies, giving rise to similar outcomes. This behaviour seems to be typical of these functionals in predicting fluorescence energies and it has been already observed in other similar ESIPT-based systems.^22^ Furthermore, the use of non-equilibrium solvation models such as corrected linear response PCM (cLR-PCM) and state specific PCM (SS-PCM) does not improve the performance of the different functionals, but rather the opposite. Overall, the mean unsigned error (MUE) values for the fluorescence energies of the enol and keto forms of HNO were 0.19 and 0.26 eV, respectively, with the LR-PCM model, 0.48–0.42 eV with cLR-PCM and 0.50–0.44 eV using SS-PCM (Tables S1 and S2 in ESI†). In a previous work,^23^ even more refined solvent models had to be used to improve the accuracy of the modelling to ESIPT dyes.

**Table 2:** Theoretical emission maxima and oscillator strength (*f*, in parentheses) calculated at B3LYP/6-31+G(d) level of theory in acetonitrile

Compound	*λ*_emission_ (nm), *f*	
	Enol S_1_	Keto S_1_
HPO	355 (0.659)	443 (0.312)
HBO	384 (1.049)	464 (0.452)
HNO	433 (0.811)	483 (0.579)

**Table 3:** Evaluation of different DFT functionals in reproducing the emission energies of HNO. Basis set employed: 6-31+G(d). Solvent model: LR-PCM

	Solvent^a^	*λ*_emission_ (nm)				
		B3LYP	CAM-B3LYP	M06-2X	PBE0	ωB97XD
Enol S_1_	Cyc	418	361	360	397	356
	DCM	429	378	377	409	372
	MeCN	433	383	382	414	377
	MeOH	433	383	381	414	377
Keto S_1_	Cyc	497	424	429	471	421
	DCM	485	429	434	464	427
	MeCN	483	431	436	463	429
	MeOH	483	431	436	463	429

a Cyc, DCM, MeCN and MeOH stand for cyclohexane, dichloromethane, acetonitrile, and methanol, respectively.

### Effect of benzannulation on the S_0_ → S_1_ excitation character

As it can be seen in Fig. 2 for HPO, molecular orbital distribution shows a HOMO mainly localized in the phenolic unit and a LUMO distributed over the heterocyclic part. This suggest that the HOMO → LUMO transition implies a considerable redistribution of electron density, as evidenced by the decrease of the contribution from the OH group to the molecular orbital and the increase of the N-acceptor contribution in oxazole in going from HOMO to LUMO. This is an important feature, since electron redistribution plays a major role in ESIPT mechanism as it sets the stage for the subsequent proton transfer.^18^

**Fig. 3:**
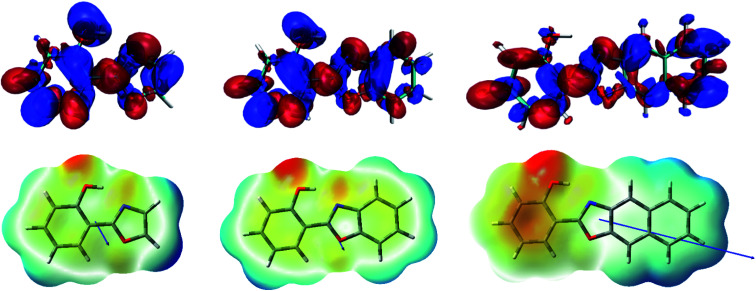
Top: density difference plots (Δ*ρ* = *ρ*_S_1__ − *ρ*_S_0__, isovalue = 0.0004) calculated with B3LYP/6-31+G(d) in acetonitrile. The blue/red zones indicate a decrease/increase of electron density upon excitation, respectively. Bottom: electrostatic potential maps and dipole moment vectors for the three compounds in acetonitrile.

In order to shed light on the nature of this process, the charge transfer (CT) character at the geometry of the E* minimum of each molecule was qualitatively examined through TDDFT calculations. The change on electron density upon excitation calculated with B3LYP/6-31+G(d) in acetonitrile is showed in Fig. 3 (top). In HPO, a visible change of density takes place on the OH group (blue lobe) together with an increase of electron density above the N atom (red lobe) in going from S_0_ to S_1_. The electron flux is mostly directed from phenol towards the heterocyclic part of the molecule, making the N atom more basic and thus favouring the subsequent proton transfer. In the case of HBO, the electron density redistribution is similar to that of HPO, although slightly less pronounced. This situation changes dramatically for HNO, as the S_0_ → S_1_ transition causes a strong polarization and thus exhibits a more marked CT character. It is important to note that the OH group of HNO plays a rather marginal role in the redistribution of electron density, in contrast to the cases of HPO and HBO. The unusual charge transfer character observed for HNO is closely related to the incorporation of an electron-rich benzene ring to the heterocyclic part of the molecule.

The electrostatic potential maps depicted in Fig. 3 (bottom) help to clarify the CT involved in the excitation to the S_1_ state of HNO. The large dipole moment of enol form in S_1_ (10.4 D) reflects the considerable polarization that takes place according to B3LYP results. Due to this solute polarization, the ESIPT reaction could experience a certain solvent-polarity induced barrier, with the consequent separation between E* and K* forms along the reaction coordinate.^24^ This effect is less likely to occur in HPO and HBO, since their dipole moments at the E* minimum in S_1_ are considerably smaller (2.6 D and 3.0 D, respectively). The distinctive behaviour of HNO*versus* its lower analogues upon light absorption could have a strong influence on ESIPT reactivity, as it is demonstrated in the following sections.

### Impact of benzannulation on the H-bond strength

**Fig. 4:**
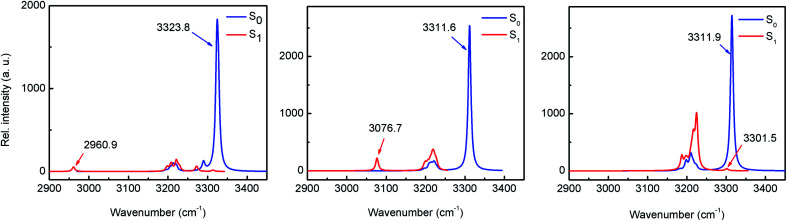
IR spectra of HPO (left), HBO (middle) and HNO (right) in the S_0_ and S_1_ states calculated with B3LYP/6-31+G(d) in acetonitrile depicting the red shifting of O–H stretching mode upon excitation.

The existence of an intramolecular H-bond between proton donor and acceptor sites is a prerequisite for ESIPT to occur.^5^ For most ESIPT systems it is well known that such an interaction becomes stronger upon photoexcitation, which leads to an almost barrierless process. The strength of the H-bond can be estimated through the computation of the IR vibrational modes corresponding to the O–H bond (specifically, the O–H stretching frequency) in either ground or excited states.^25^ The results of the computational calculations for the enol forms of HPO, HBO and HNO reveal different behaviours for each one of them (Fig. 4). In HPO, the O–H stretching is red-shifted by 363.8 cm^−1^ upon excitation, from 3323.8 cm^−1^ to 2960.9 cm^−1^, which provides evidence for the O–H⋯N bond enhancement in S_1_. In the case of HBO this effect is slightly smaller, with a red shift of 234.9 cm^−1^. Surprisingly, in HNO the enhancement of H-bond in S_1_ state is rather negligible, as the change in the O–H vibrational frequency in going from S_0_ to S_1_ is only 10.4 cm^−1^. These results concur with those obtained from NCI (non-covalent interaction)^26^ and QTAIM analysis^27,28^ (see ESI for details†). The reason why the H-bond in HPO, HBO (and many other related systems^29^) becomes stronger in S_1_ state owes to the fact that the electron density distribution over the N atom in S_1_ is greater than in ground state, making that nitrogen more basic (Fig. 3). In contrast to these cases, in HNO the electron density moves away from N atom upon excitation due to the aforementioned larger extent of charge transfer. Therefore, in S_1_ the N acceptor of HNO is at most as basic as in S_0_, which should result in a similar proton-transfer reactivity in both electronic states. To confirm this hypothesis, it is necessary to model the potential energy surfaces in S_0_ and S_1_ states through DFT and TDDFT calculations, respectively.

### Effect of the extension of oxazole π-conjugation on ESIPT reaction profile

**Fig. 5:**
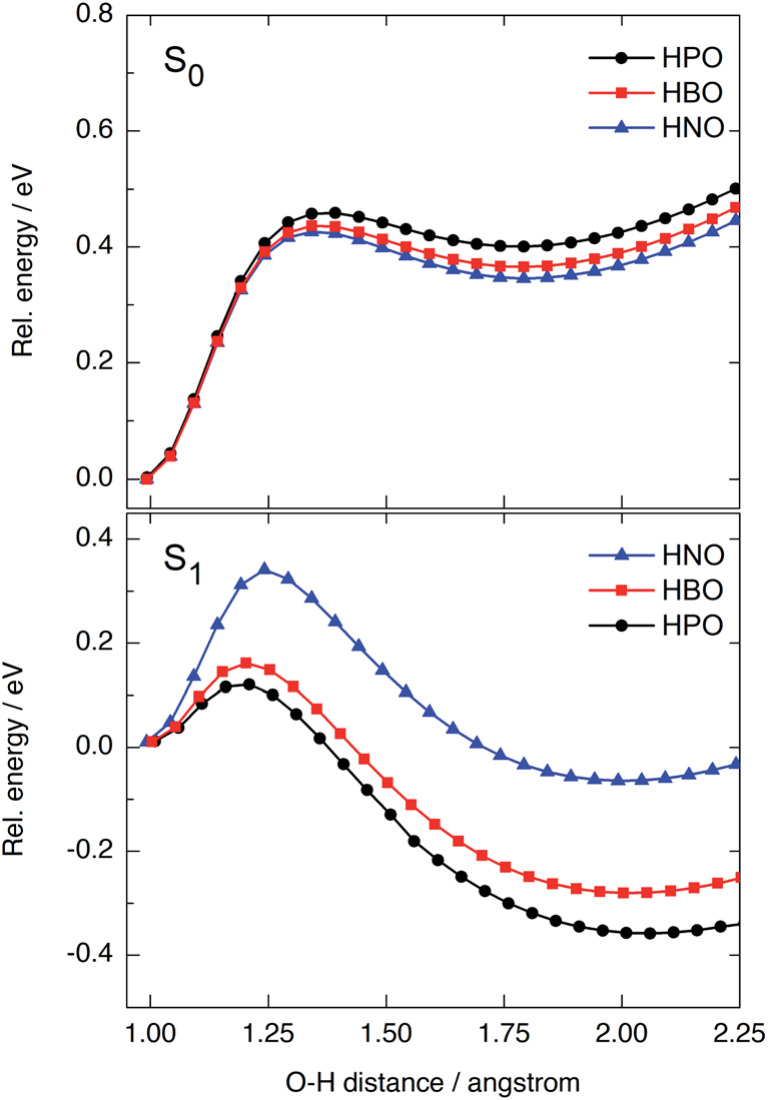
PES calculated at B3LYP/6-31+G(d) level in acetonitrile for HPO (black), HBO (red) and HNO (blue) in the ground (top) and excited (bottom) states. For each curve, the energies are relative to the energy of the corresponding adiabatic enol minimum.

We have modelled the ESIPT process for HPO, HBO and HNO using DFT and TDDFT computational calculations at the B3LYP/6-31+G(d)/IEFPCM level of theory. In ground state, the energy barrier for tautomerization (E → K) is quite high in all cases (0.42–0.46 eV, Fig. 5, top), which rules out the presence of the keto form in the S_0_ state as one of the light-absorbing species at 298 K. The ground-state potential energy surface (PES) of HNO (Fig. 5, top, in blue) lies at slightly lower energies than that of HBO (red) and HPO (black), indicating the S_0_ K tautomer of HNO is somewhat more stable than the others. This makes sense if one considers that a naphthoxazole unit is richer in electrons than benzoxazole or oxazole moieties, and therefore it can accept a proton more easily. As there is a large energy difference between enol and keto forms in ground state, the back-proton transfer exhibits low barrier.

Vertical excitation of enol form to the Franck–Condon (FC) region of S_1_ state is at 4.02 eV in HPO, 3.77 eV in HBO and 3.41 eV in HNO above ground-state PES (Fig. S9 in ESI†). The state decays to the local E* minimum on S_1_ state, which lies almost ∼0.25–0.27 eV below the FC level in the three cases. The energy barrier for proton transfer in S_1_ increases with each successive benzannulation, from 0.11 eV (2.5 kcal mol^−1^) in HPO to 0.16 eV (3.7 kcal mol^−1^) in HBO, to 0.33 eV (7.6 kcal mol^−1^) in HNO (Fig. 5, bottom). Note that for HNO, the proton transfer barrier in S_1_ is comparable to that one in S_0_ (0.42 eV) which can favour a radiative decay channel from E* to the ground state. Additionally, the K* tautomer of HNO is almost degenerate with the E* form, being the energy difference of only 0.08 eV (Fig. S9†), which implies a lower thermodynamic driving force for proton transfer.

Besides fluorescence emission, a possible deactivation channel from K* form in S_1_ implies the adiabatic back-proton transfer to recover the E* tautomer on the S_1_ PES by surmounting the reverse barrier. This barrier is estimated to be 0.50 eV for HPO, 0.46 eV for HBO and 0.40 for HNO at B3LYP/6-31+G(d) level in acetonitrile. As the K* form of HNO is higher in energy (relative to HNO-E*) than the K* form of the other dyes, it is easier for HNO to undergo back-proton transfer in excited state.

In summary, benzannulation of the oxazole core leads to higher energy barriers for ESIPT in the first singlet excited state and to a less stable K* tautomer, as a result of the modification of charge transfer properties. Nevertheless, the structural modification has little impact on ESIPT emission energies, unlike the observed for other benzazoles benzannulated at the phenol side.

### Rationalization of the results under the light of Baird's rule

**Fig. 6:**
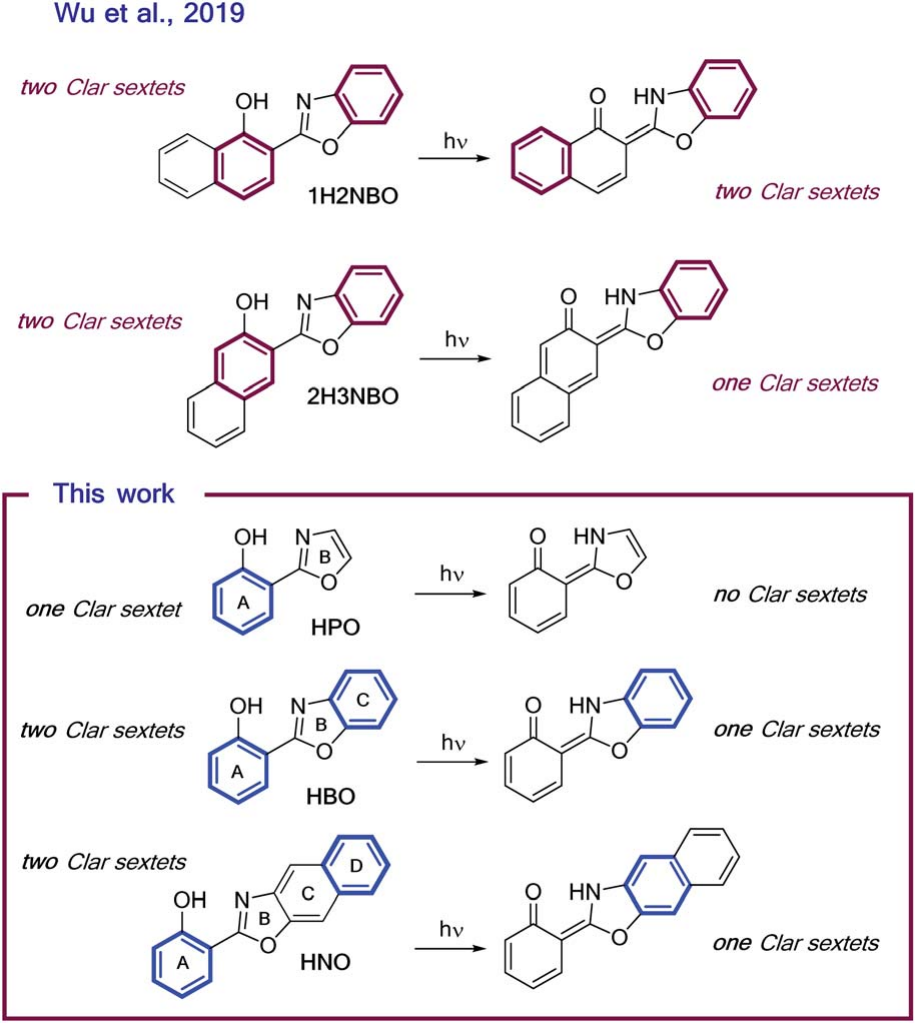
Schematic illustrations of ESIPT in (A) 1H2NBO and 2H3NBO and (B) HPO, HBO and HNO. Clar's sextets are highlighted in bold.

Recently, the “anomalous” emissive behaviour of the benzannulated HBO derivatives 1H2NBO and 2H3NBO (Fig. 6) was rationalized in terms of the Baird's rule.^13,14^ Despite these compounds are structurally similar, they exhibit very different ESIPT emission with a maximum at 470 nm for 1H2NBO and at 670 nm in the case 2H3NBO. Considering the Baird's rule, ESIPT provides a way to get rid of Baird antiaromaticity by transferring a proton in the S_1_ state for these compounds. Thus, the emission maximum of K* form of 1H2NBO and 2H3NBO varies depending on the antiaromaticity of the K* species formed. By combining Baird's rule^15^ with Clar's aromatic π-sextet rule,^30^ Wu *et al.* suggested that the relaxation of K* to the hot ground state relieves more antiaromaticity in 1H2NBO than in 2H3NBO, as the latter is considerably less antiaromatic in S_1_.^14^ The enol form of 1H2NBO in S_1_ exhibits two complete antiaromatic Clar's sextets that remains unchanged upon tautomerization, whereas in 2H3NBO the formation of the keto isomer implies the loss of one of those sextets (Fig. 6). As a consequence, the keto form of 2H3NBO is less antiaromatic (*ergo* more stable) than the one of 1H2NBO in S_1_, and therefore its fluorescence emission results red-shifted.

On the other hand, a cursory look on the structures of HPO, HBO and HNO reveals that the excited-state stabilization of these fluorophores should proceed with similar extent, since ESIPT could alleviate the antiaromaticity of only one Clar's sextet in all cases (Fig. 6). To validate this intuitive hypothesis, it is necessary to provide a quantitative estimation of excited-state aromaticity. The evaluation of aromaticity can be done by using different structural,^31^ energetic,^32^ magnetic,^33^ electronic^34^ and reactivity-based^35^ descriptors. In the present case we have chosen the dissected nucleus-independent chemical shifts, NICS(1)_*zz*_, analysis in order to provide a comparison with the results of Wu *et al.* According to NICS(1)_*zz*_ analysis, aromatic compounds are characterized by large, negative values due to magnetic shielding induced by the diatropic ring current. On the other hand, antiaromatic rings exhibit paratropic current, which causes deshielding at the ring and thus affords positive NICS(1)_*zz*_ values.

In the ground state the NICS(1)_*zz*_ analysis predicts that all the rings (A–D, Fig. 6) of the enol form of HPO, HBO and HNO are strongly aromatic. In HPO, both A–B rings are almost equally aromatic, whereas in HBO the C ring is the most aromatic one. Curiously, C is also the most aromatic ring in HNO.

Upon photoexcitation, the E* form becomes globally antiaromatic in all cases, with NICS(1)_*zz*_ values of +29.1, +28.3 and +48.7 ppm for HPO, HBO and HNO, respectively. The A ring in HPO and HBO turns largely antiaromatic in S_1_, as Baird's rule predicts. Interestingly, the A ring in HNO remains slightly aromatic in S_1_, whereas C and D rings exhibit considerable paratropicity. The relief of antiaromaticity, which is believed to stabilize the K* form, occurs mainly from the A ring in HPO and HBO but from the C and D rings in HNO. This results demonstrate, again, that HNO behaves differently than its lower analogues in excited state.

**Fig. 7:**
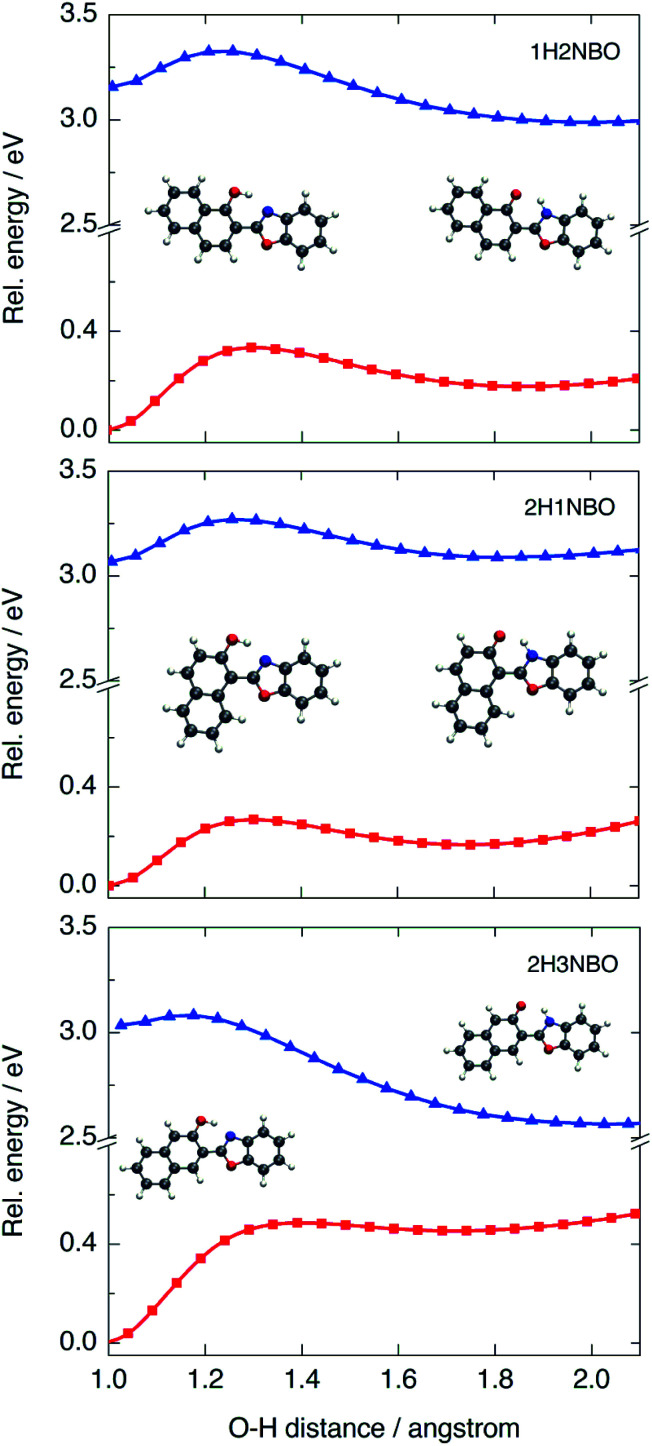
PES calculated at B3LYP/6-31+G(d) level in acetonitrile for the three naphthol-derivatives 1H2NBO, 2H1NBO and 2H3NBO in the S_0_ (red) and S_1_ (blue) states.

To determine the origin of the particular fluctuation of aromaticity observed in the ESIPT of HNO, the NICS(1)_*zz*_ analysis was performed to the naphthol-containing derivatives 1H2NBO, 2H3NBO^9,14^ and 2H1NBO^12,13^ (Scheme 2 and Fig. 7). Since the three compounds have the same number of rings than HNO, the comparison becomes straightforward. The resulting values are listed in Table 5.

The global NICS(1)_*zz*_ value for the ground-state E tautomer is around −90 ppm in all cases, and *ca.* −70 ppm for the K form. However, this trend changes significantly upon excitation. At the E* geometry, 2H3NBO is markedly more antiaromatic than 1H2NBO and 2H1NBO by 26.3 ppm and 41.1 ppm, respectively. The origin of this difference seems to be related with the paratropicity of the naphthol subunit: in 2H3NBO, the local NICS(1)_*zz*_ value of naphthol is 95.1 ppm, whereas in 1H2NBO and 2H1NBO is 57.4 and 38 ppm, respectively. This is a noteworthy aspect: while the Baird's rule predicts the naphthol system to be antiaromatic in the first singlet excited state (as suggested by Lampkin *et al.* using naphthalene as model^13^), the degree of antiaromaticity appears to be strongly dependent on the orientation of naphthol with respect to the rest of the molecule in the studied examples. As a consequence, it is difficult to explain why the 2H3NBO derivative becomes more antiaromatic than the others on the sole basis of this rule.

Computed *Δ*NICS(1)_*zz*_ (K*–K) for 2H3NBO (*Δ* = 63.7 ppm), 1H2NBO (*Δ* = 71.2 ppm) and 2H1NBO (*Δ* = 78.1 ppm) could give an idea of the relief of antiaromaticity that accompanies fluorescence emission (670 nm, 460 nm and 470 nm in hexane, respectively), as Wu *et al.* proposed.^14^ However, there seems to be no lineal relationship between calculated *Δ*NICS(1)_*zz*_ and fluorescence energy in a strict way. 1H2NBO relieves more antiaromaticity than 2H3NBO (*ca.* +7.5 ppm) in going from K* to K and its fluorescence emission is shorter than the one of 2H3NBO by 200 nm. At the same time, 2H1NBO relieves more antiaromaticity than 1H2NBO (+6.9 ppm), but the fluorescence maxima of both compounds are very close among each other (460 *vs.* 470 nm). So, a similar relief of antiaromaticity is associated with a huge red shift in emission in one case but not in another. To fully understand this discrepancy, it is necessary to go beyond Baird's rule and consider the ESIPT process further.

The PES modelled at the S_0_ and S_1_ states for the proton-transfer process in 1H2NBO, 2H1NBO and 2H3NBO are shown in Fig. 7. 1H2NBO and 2H1NBO exhibit similar energetic profiles, although they differ in that TDDFT predicts the ESIPT reaction of 1H2NBO to be endergonic by *ca.* 0.02 eV. Lampkin *et al.* attributed this endergonicity to an error associated with the geometry optimization of K*,^13^ but it seems rather to be a typical feature of the ESIPT compounds in which an extra fused ring is located at a non-favourable position.^36^ Furthermore, in some cases CASSCF calculations also predicts endergonic ESIPT, in the same way as TDDFT does.^37^ In any case, it is undeniable that ESIPT in 2H3NBO is far more exothermic than in the other cases. This behaviour is consistent with the results of NICS(1)_*zz*_ analysis. The large stabilization of K* in 2H3NBO can be interpreted as a result of the considerable relief of antiaromaticity associated with the E* → K* photoisomerization (*Δ*NICS(1)_*zz*_ = 66.9 ppm), much larger than that of 1H2NBO (28.8 ppm) and 2H1NBO (10 ppm). As can be seen in Fig. 7, the red-shifted emission of 2H3NBO is a consequence of the proximity of both the S_1_ and the S_0_ surfaces. The high energy of the K form in ground state can be understood in terms of the Clar's rule, since its formation implies the loss of one aromatic Clar's sextet. Unlike this case, the K tautomer in 1H2NBO and 2H1NBO preserves the Clar's sextet of the naphthol system after isomerization, which makes it logically more stable in S_0_ and leads to a larger S_0_–S_1_ energy gap. These observations are a clear example of the complementarity between Baird's and Clar's rules applied to ESIPT.

Finally, we can compare the results of the NICS(1)_*zz*_ analysis in order to provide a complete description of the effect of benzannulation on ESIPT in terms of excited state aromaticity. If we compare the local changes in aromaticity that take place during ESIPT, the behaviour of HNO results very different to that of the naphthol-containing dyes 1H2NBO, 2H1NBO and 2H3NBO. While in this series the naphthol subunit becomes antiaromatic upon light absorption, in HNO the rise of antiaromaticity occurs at the heterocyclic part of the molecule. Moreover, the phenol ring in the E* form of HNO does not exhibit paratropicity at all in the S_1_ state, despite being a Clar's sextet that should be antiaromatic according to the Baird's rule. The same applies for the benzoxazole moiety of 1H2NBO, 2H1NBO and 2H3NBO, which never becomes locally antiaromatic in S_1_. In summary: the fluctuation pattern of excited state aromaticity in HNO appears to be the opposite of that in the naphthol-containing series.

One possible explanation for this odd behaviour is that charge transfer in S_1_ can actually modulate the aromaticity of different regions within the same molecule. As we have detailed before in this text, in HNO the vertical transition to the first singlet excited state exhibits a considerable CT character which increases the electron density of the phenol ring. This increment is connected with the small but negative NICS(1)_*zz*_ value of that ring in the E* form of HNO (−3.9 ppm, Table 4), which seems to indicate that CT avoids the ring to become antiaromatic. At the same time, π depletion of C–D rings of HNO makes them markedly antiaromatic in S_1_. This trend is reverted as ESIPT occurs, since it implies the translocation of a proton and an electron towards the heterocyclic moiety (see Fig. S11 in ESI†). At the K* geometry, the CT direction has been reverted and the C–D systems are no longer antiaromatic but, at the same time, the A ring has become antiaromatic in turn.

**Table 4:** NICS(1)_*zz*_ values (in ppm) for enol and keto forms of HPO, HBO and HNO in the S_0_ and S_1_ states^a^

Dye	S_0_		S_1_	
		Global *Δ*NICS(1)_*zz*_^b^		Global *Δ*NICS(1)_*zz*_
HPO				
Enol	**−44.6** (−21.6, −23.0)	**0**	**29.1** (36.9, −7.8)	**73.7**
Keto	**−31.5** (−13.4, −18.2)	**13.1**	**10.7** (16.6, −5.8)	**55.3**

HBO				
Enol	**−66.7** (−20.8, −18.1, −27.8)	**0**	**28.3** (26.3, 0.1, 1.8)	**95.0**
Keto	**−52.3** (−12.5, −13.1, −26.8)	**14.4**	**−5.3** (12.1, −2.6, −14.8)	**61.4**

HNO				
Enol	**−92.4** (−20.5, −15.5, −28.9, −27.5)	**0**	**48.7** (−3.9, 1.3, 34.3, 16.9)	**141.1**
Keto	**−75.9** (−11.3, −10.4, −26.6, −27.6)	**16.5**	**−19.8** (10.1, −4.3, −9.5, −16.1)	**72.6**

a NICS(1)_*zz*_ values were calculated at PW91/IGLOIII level of theory from the geometries optimised at ωB97X-D/6-311+G(d,p) level. For each compound, the individual values for A–D rings (in parentheses) are informed from left to right. The more negative (positive) the value, the more aromatic (antiaromatic) the character.

b Variation with respect to the NICS(1)_*zz*_ value of the ground-state enol form, for each molecule.

The same analysis can be applied for 2H3NBO, which is the only dye of the naphthol series exhibiting a significant degree of CT in the S_1_ state (see Fig. S12 in ESI†). As it was previously discussed, the E* tautomer of 2H3NBO shows high paratropicity at the π-depleted naphthol unit and significant diatropicity at the rest of the molecule. Since the CT direction (naphthol-to-benzoxazole) does not change during ESIPT in 2H3NBO, the aromatic character of benzoxazole remains almost unaltered during E* → K* isomerization. Within the series, the varying CT character finely tunes the aromaticity of the rings: the higher the electron density gained, the more aromatic the system and more stable the K* tautomer (compare Table 5 and Fig. S12†). This could explain both the deeply exothermic ESIPT in 2H3NBO and also the endergonic one in 2H1NBO.

**Table 5:** NICS(1)_*zz*_ values (in ppm) for enol and keto forms of 1H2NBO,^a^2H3NBO^a^ and 2H1NBO^b^ in the S_0_ and S_1_ states

Dye	S_0_		S_1_	
		Global *Δ*NICS(1)_*zz*_^c^		Global *Δ*NICS(1)_*zz*_^c^
1H2NBO				
Enol	**−90.4** (−26.2, −19.4, −17.4, −27.4)	**0**	**30.8** (21.5, 35.9, −11.0, −15.6)	**121.2**
Keto	**−69.2** (−25.2, −7.3, −10.9, −25.8)	**21.2**	**2.0** (5.8, 12.3, −2.9, −13.2)	**92.4**

2H3NBO				
Enol	**−91.3** (−23.9, −22.3, −17.6, −27.5)	**0**	**57.1** (38.3, 56.8, −15.1, −22.8)	**148.4**
Keto	**−73.5** (−15.7, −16.7, −14.0, −27.1)	**17.8**	**−9.8** (−0.1, 16.5, −5.8, −20.4)	**81.5**

2H1NBO				
Enol	**−90.2** (−26.6, −18.3, −17.9, −27.4)	**0**	**16.0** (8.9, 29.1, −9.2, −12.9)	**106.2**
Keto	**−71.5** (−24.9, −8.1, −12.3, −26.3)	**18.7**	**6.6** (8.1, 14.2, −3.7, −12.0)	**96.8**

a Extracted from ref. 14.

b Calculated at PW91/IGLOIII level of theory from the geometries optimised at ωB97X-D/6-311+G(d,p) level.

c Variation with respect to the NICS(1)_*zz*_ value of the ground-state enol form, for each molecule.

From what precedes, charge transfer arises as an essential aspect that must be considered for a complete rationalization of the impact of benzannulation on ESIPT. In a more general sense, ESIPT can be facilitated by the combination of two effects: on the one hand, the antiaromaticity alleviation caused by CT in the S_1_ state and on the other hand, the basicity enhancement of the proton-acceptor site caused by the electron redistribution. When light absorption triggers CT in an unfavourable direction (as in HNO), the subsequent ESIPT becomes hampered due to the lack of driving force.

In agreement with the reported by Wu *et al.*, ESIPT provides a way to avoid antiaromaticity in excited state, but mostly because of the effect of electron redistribution. The exact mechanism by which CT modulates the excited-state aromaticity/antiaromaticity of a ring is not fully understood at this stage, but further work could lead to a comprehensive knowledge of the phenomenon.

## Conclusions

The results presented in this study demonstrated that the ESIPT reactivity is significantly affected by the size of the π-conjugated framework. Unlike previous cases, benzannulation at the heterocyclic part of HPO does not lead to a significant red shift in ESIPT emission. Moreover, benzannulation of HBO to give HNO causes a remarkable change in the charge transfer character of the S_0_ → S_1_ excitation that leads to a redistribution of electron density from the naphthoxazole subunit to the phenol ring. As a consequence, there is a large barrier for ESIPT in the S_1_ state. The extension of conjugation also decreases the basicity of the N acceptor and weakens the intramolecular O–H⋯N bond, essential for ESIPT to occur.

On the other hand, benzannulation of HBO at the phenol unit leads to a very large redshift in emission only when it occurs at a specific site of the molecule, as proposed by Nagaoka *et al.* This particular array allows to maximize the CT extent in the S_1_ state.

The results presented herein can be interpreted using the Baird's rule and the Clar's rule of sextets. Nevertheless, the local variations of aromaticity observed for individual rings in the S_1_ state cannot be explained satisfactorily by using these approaches only. The analysis of local NICS(1)_*zz*_ fluctuation during the ESIPT process becomes meaningful when the CT character of the S_1_ state is considered. This observation seems to suggest that CT in S_1_ is actually able to alleviate the local Baird antiaromaticity in ESIPT compounds, providing a driving force for the process. The impact of CT on the excited-state aromaticity requires detailed theoretical and experimental work, which will be the focus of future work.

## Materials and methods

All reagents and solvents were obtained from Sigma Aldrich and used as received. Spectroscopic grade solvents were used for UV-vis absorption and fluorescence spectra measurements. UV-visible spectra of the compounds in solution were recorded with a Shimadzu UV-1800 Spectrophotometer at 25 °C. Fluorescence spectra of the samples were recorded with an Agilent Cary Eclipse Fluorescence Spectrophotometer at 25 °C. ^1^H NMR and ^13^C NMR were recorded on a 400 MHz Bruker nuclear magnetic resonance spectrometer.

### Synthesis

HNO was synthesized according to a reported protocol,^38^ by reaction of 355 mg (2.2 mmol) of 3-amine-2-naphthol with 370 mg (2.7 mmol) of salicylic acid in 10 mL of polyphosphoric acid. The reaction mixture was heated to 180 °C for 3 h and then poured into ice water and neutralized with NaHCO_3_. The solid was filtered, washed with water and purified by column chromatography using hexane/ethyl acetate 100 : 0 to 80 : 20 as eluent.

#### 2-(Naphtho[2,3-*d*]oxazol-2-yl)phenol (HNO)^38^

White solid. Yield: 28%. ^1^H NMR (400 MHz, (CDCl_3_, 25 °C)): *δ* 11.58 (s, 1H), 8.16 (s, 1H), 8.09 (dd, *J* = 7.9, 1.4 Hz, 1H), 7.94–8.04 (m, 3H), 7.56–7.45 (m, 3H), 7.15 (d, *J* = 8.3 Hz, 1H), 7.04 (t, *J* = 7.5 Hz, 1H) ppm. ^13^C NMR (101 MHz, CDCl_3_) *δ* 165.1, 159.6, 148.2, 140.0, 134.3, 131.9, 131.8, 128.6, 128.1, 127.8, 125.9, 125.2, 119.8, 117.7, 116.7, 110.5, 106.7 ppm.

### Computational methods

All DFT and TDDFT calculations were performed using the Gaussian 09 program.^39^ The relevant stationary points were fully optimized using the B3LYP functional with the 6-31+G(d) basis set, although CAM-B3LYP, M06-2X, PBE0 and ωB97XD functionals were also used in certain cases for comparative purposes. The nature of the obtained stationary points was verified by Hessian diagonalization and harmonic frequency analyses. Solvent effects were included using three different formalisms: linear response PCM (LR-PCM), corrected linear response PCM (cLR-PCM) and state specific PCM (SS-PCM) with non-equilibrium solvation. Relaxed scans were computed by allowing all the internal degrees of freedom to relax apart from the driving coordinate (O–H distance, step length = 0.05 Å). Vertical excitation and emission energies were calculated within the linear response scheme of TDDFT. For NCI and QTAIM analyses the Multiwfn software was used.^40^ The dissected nucleus-independent chemical shifts (NICS(1)_*zz*_) were calculated at 1 Å above the different rings taking into account only contributions from the out-of-plane tensor component perpendicular to the ring planes. NICS(1)_*zz*_ values were computed using the PW91 functional with the IGLOIII basis set. For the estimation of NICS(1)_*zz*_ in the S_1_ state, NICS calculations were performed as open-shell triplet states employing the geometries optimized at the S_1_ state as reported by Wu *et al.*^14^ Visualization and graphics rendering were carried out with GaussView 5.0.8 (ref. 41) and VMD 1.9.3.^42^

## Conflicts of interest

There are no conflicts to declare.

## Supplementary Material

RA-010-D0RA05802E-s001
