# Supplementary material for: Impact of benzannulation on ESIPT in 2-(2′-hydroxyphenyl)-oxazoles: a unified perspective in terms of excited-state aromaticity and intramolecular charge transfer
Source: RSC Adv. 2020 Oct 23;10(64):39049–59. doi: 10.1039/d0ra05802e (PMC9057364; doi:10.1039/d0ra05802e)
Supplement: RA-010-D0RA05802E-s001 [file RA-010-D0RA05802E-s001.pdf]

Electronic Supplementary Material (ESI) for RSC Advances.
This journal is © The Royal Society of Chemistry 2020

SUPPORTING INFORMATION

Impact of benzannulation on ESIPT in 2-(2'-hydroxyphenyl)-oxazoles: a unified perspective in terms of excited-state aromaticity and intramolecular charge transfer

Leandro D. Mena,[a]* D. M. A. Vera[b] and María T. Baumgartner[a]*

[a] INFIQC, Departamento de Química Orgánica, Facultad de Ciencias Químicas, Universidad Nacional de Córdoba, Ciudad Universitaria, X5000HUA, Córdoba, Argentina.
[b] QUIAMM-INBIOTEC- Departamento de Química Facultad de Ciencias Exactas y Naturales, Universidad Nacional de Mar del Plata Mar del Plata, Argentina
** Corresponding authors: L. D. M.: lmena@fcq.unc.edu.ar, M. T. B.: tere@fcq.unc.edu.ar*

Contents

1.	^{1}H and ^{13}C NMR spectra of **HNO**	3
2.	MOs involved in $S_0 \rightarrow S_1$ excitation	4
3.	Emission energies for **HNO** (keto) computed with LR-PCM, cLR-PCM and SS-PCM models	5
4.	Non- covalent interactions (NCI) analysis	5
5.	QTAIM analysis	7
6.	Energy levels	8
7.	Relevant information about stationary points	9
8.	Optimised coordinates for all compounds	10

1. ^{1}H and ^{13}C NMR spectra of HNO

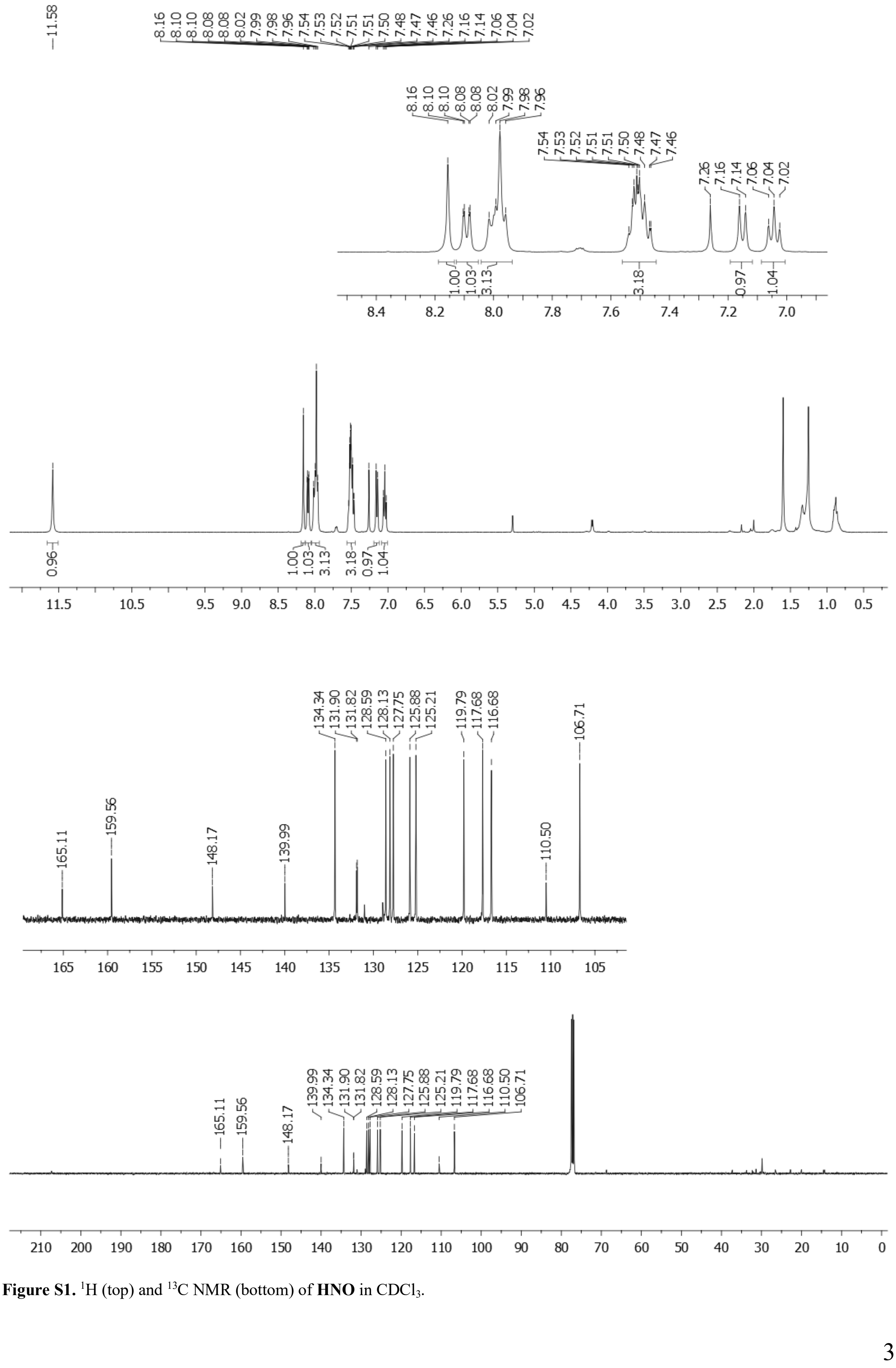

Figure S1. ^{1}H (top) and ^{13}C NMR (bottom) of **HNO** in $CDCl_3$.

2. *MOs involved in $S_0 \rightarrow S_1$ excitation*

a) **HPO**

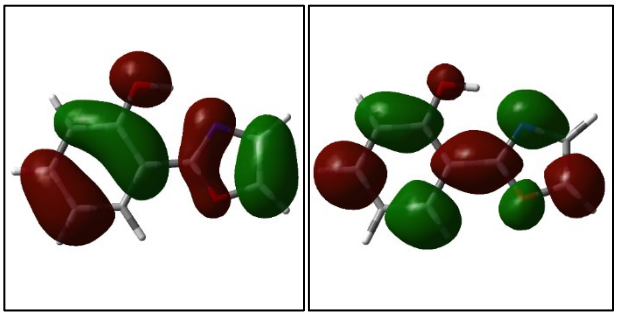

Figure S2. HOMO (left) and LUMO (right) of **HPO** in the S_0 state.

Excited State 1: Singlet-A 4.0217 eV
308.29 nm f=0.5103 <S**2>=0.000
42 -> 43 0.69245

b) **HBO**

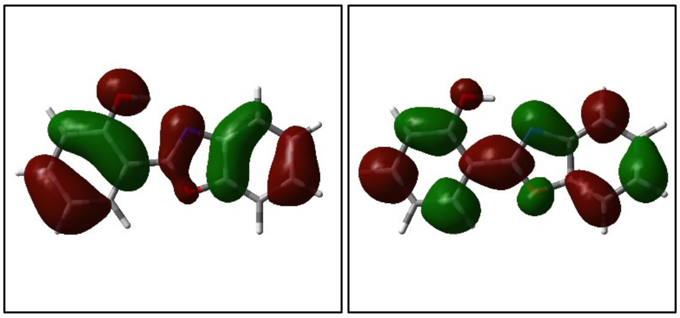

Figure S3. HOMO (left) and LUMO (right) of **HBO** in the S_0 state

Excited State 1: Singlet-A 3.7671 eV
329.12 nm f=0.8517 <S**2>=0.000
55 -> 56 0.69938

c) **HNO**

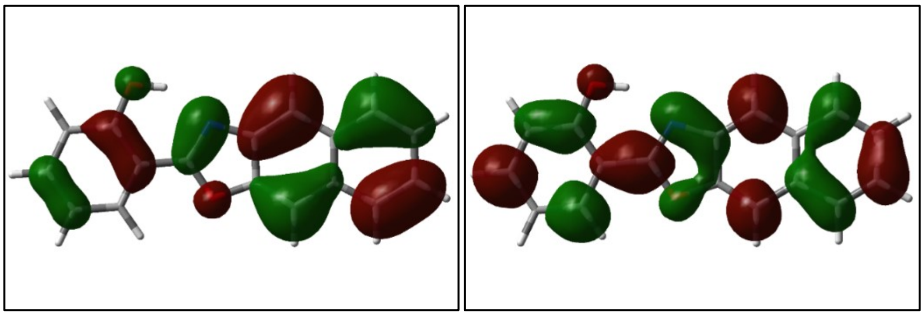

Figure S4. HOMO (left) and LUMO (right) of **HNO** in the S_0 state

Excited State 1: Singlet-A
3.4113 eV 363.45 nm f=0.6372 <S**2>=0.000
68 -> 69 0.69574

3. *Emission energies for HNO (keto) computed with LR-PCM, cLR-PCM and SS-PCM models*

Table S1 Emission energies of **HNO** (in eV) calculated using different functionals with continuum solvation models for acetonitrile. The error with respect to the experimental fluorescence energies are presented in parentheses. MUE: mean unsigned error (in eV).

	B3LYP	CAM-B3LYP	M06-2X	PBE0	ωB97XD	MUE
			enol form			
LR-PCM	2.86 (-0.13)	3.23 (0.24)	3.25 (0.26)	3.00 (0.01)	3.29 (0.30)	0.19
cLR-PCM	3.69 (0.70)	3.49 (0.50)	3.50 (0.51)	3.09 (0.10)	3.56 (0.57)	0.48
SS-PCM	2.03 (-0.96)	3.19 (0.20)	3.1 (0.11)	2.23 (-0.76)	3.45 (0.46)	0.50
			keto form			
LR-PCM	2.57 (0.06)	2.88 (0.37)	2.84 (0.33)	2.68 (0.17)	2.89 (0.38)	0.26
cLR-PCM	2.65 (0.14)	3.07 (0.56)	3.04 (0.53)	2.78 (0.27)	3.09 (0.58)	0.42
SS-PCM	2.16 (-0.35)	3.13 (0.62)	3.04 (0.53)	2.41 (-0.11)	3.11 (0.60)	0.44

Table S2. Emission energies of **HNO** (in eV) calculated using different functionals with continuum solvation models for cyclohexane. The error with respect to the experimental fluorescence energies are presented in parentheses. MUE: mean unsigned error (in eV).

	B3LYP	CAM-B3LYP	M06-2X	PBE0	ωB97XD	MUE
			enol form			
LR-PCM	2.96	3.43	3.44	3.12	3.49	-
cLR-PCM	2.91	3.53	3.54	3.12	3.59	-
SS-PCM	2.63	3.51	3.51	2.87	3.59	-
			keto form			
LR-PCM	2.49 (-0.02)	2.92 (0.41)	2.89 (0.38)	2.63 (0.12)	2.95 (0.44)	0.27
cLR-PCM	2.47 (-0.04)	2.99 (0.48)	2.96 (0.45)	2.63 (0.12)	3.02 (0.51)	0.31
SS-PCM	2.29 (-0.22)	2.99 (0.48)	2.95 (0.44)	2.51 (-0.00)	3.03 (0.51)	0.33

4. *Non- covalent interactions (NCI) analysis*

For the analysis of the OH--π interaction the RDG function has been used

$$\boldsymbol{RDG}\,(r) = \frac{1}{2(3\pi^2)^{1/3}} \frac{|\nabla\rho(r)|}{\rho(r)^{4/3}} \qquad (1)$$

where ρ(r) is the total electron density and the RDG (r) is the reduced density gradient of the exchange contribution. According to Atoms in Molecules (AIM) theory the nature of a weak interaction depends on both the λ_2 eigenvalue and the electron density:

$$\Omega\,(r) = \textbf{Sign}\,(\lambda_2(r))\rho(r) \qquad (2)$$

When $\lambda_2>0$ the interaction is bonding, and the opposite if $\lambda_2<0$. In Fig. **S9-S11** the scatter plots of RDG function vs. Ω(r) function are shown for **HPO, HBO** and **HNO** in the S_0 and S_1 states. It is important to note that the left spike (marked with red circle) shifts to

the left in S_1 in the case of **HPO** and **HBO**, indicating a strengthening in the OH−π interaction upon excitation, whereas remains almost unaltered in **HNO**.

a) **HPO**

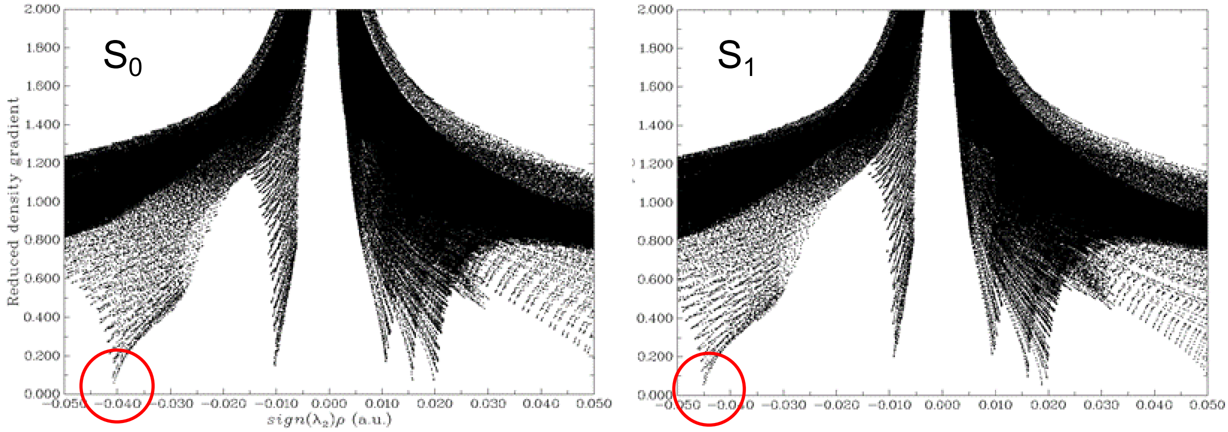

Figure S5 RDG scatter plots (isovalue=0.6) in S_0 and S_1. The left spike correspond to the attractive OH-*π* interaction.

b) **HBO**

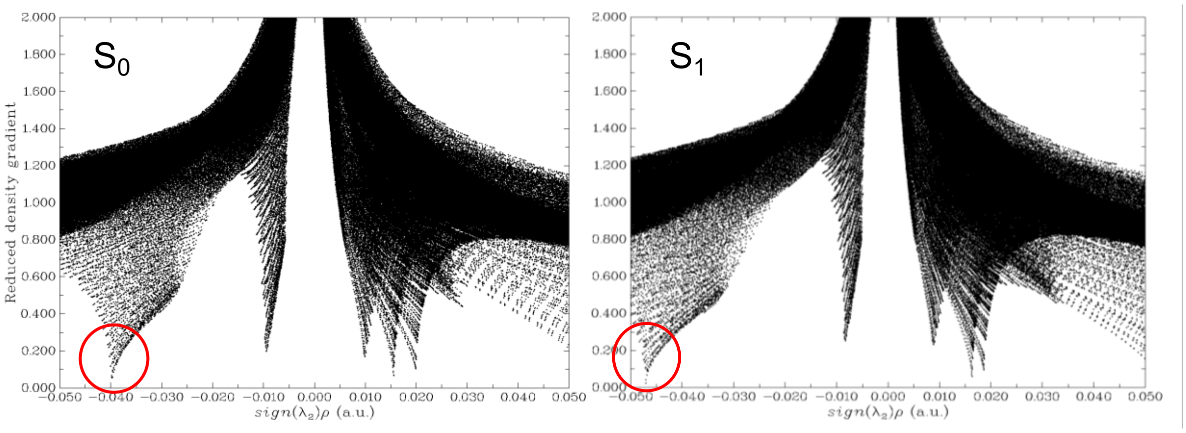

Figure S6 RDG scatter plots (isovalue=0.6) in S_0 and S_1. The left spike correspond to the attractive OH-*π* interaction.

c) **HNO**

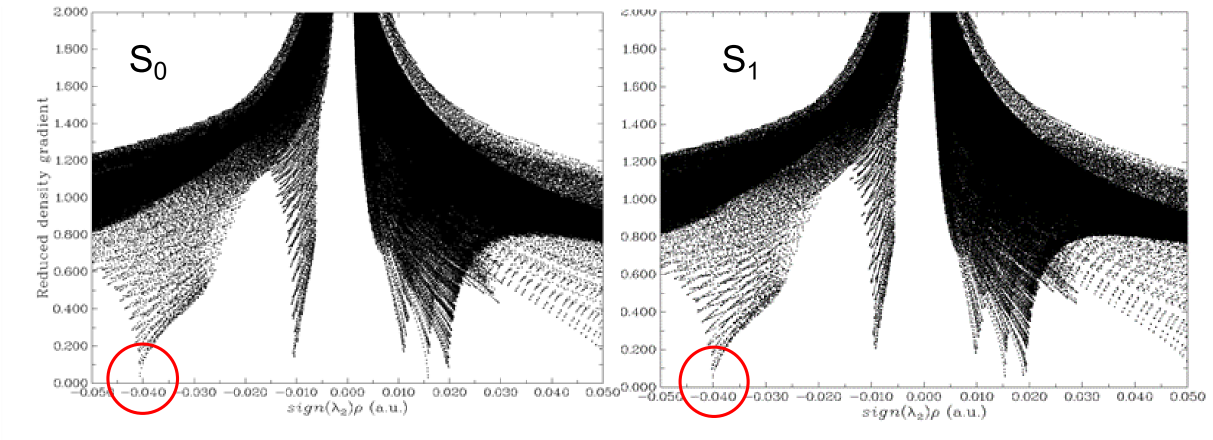

Figure S7 RDG scatter plots (isovalue=0.6) in S_0 and S_1. The left spike correspond to the attractive OH-*π* interaction.

5. *QTAIM analysis*

According to the QTAIM approach, a bonding interaction is related to the existence of a path of maximum electron density ρ(r) (the bond path) connecting the corresponding atomic basins. A *bond critical point* (BCP) is a point along this bond path at the interatomic surface where ρ(r) reaches a minimum value. In the present case, we analysed the topology of ρ(r), looking for the presence of a BCP between the hydrogen atom and the N acceptor, and then we calculated ρ(r), its Laplacian, $\nabla^2\rho$(r) and the potential energy density *V(r)* at that BCP. According to the equation proposed by Espinosa *et al.* [i], the H-bond energy can be estimated from *V(r)* as follows:

$$E_{HB} = \frac{1}{2} V(r) \quad (1)$$

Topological analysis revealed the existence of strong hydrogen bonds in all compounds in both S_0 and S_1 states (Table 5 in main text). (Note: A H-bond is defined as "strong" when $\rho(r) \geq 0.03$) [ii]. Good correlations were found between the H-N distance and ρ(r), $\nabla^2\rho$(r), total electron energy density *H(r)* and H-bond energy (Fig. S8). According to QTAIM analysis results, the H-bond in **HPO** results considerably strengthened by 2.05 kcal/mol in S_1, whereas in **HBO** the interaction increases its intensity in 1.17 kcal/mol. Unlike these cases, photoexcitation weakens the intramolecular H-bond of **HNO** by 0.18 kcal/mol, in agreement with the observed in IR calculations and NCI analysis.

Table S3 Selected parameters related to H-bond for studied the *enol* forms of the studied compounds obtained from QTAIM analysis

Dye/state		H-N distance (Å)	ρ(r), a.u.	$\nabla^2\rho$(r), a.u.	H-bond energy[a]	ΔE H-bond $S_0 \rightarrow S_1$[a]
**HPO**	S_0	1.8093	0.0399	0.1178	10.18	**2.05**
	S_1	1.7317	0.0471	0.1341	12.23	
**HBO**	S_0	1.8003	0.0407	0.1201	10.50	**1.17**
	S_1	1.7552	0.0451	0.1292	11.64	
**HNO**	S_0	1.8008	0.0407	0.1195	10.45	**-0.18**
	S_1	1.8046	0.0401	0.1184	10.26	

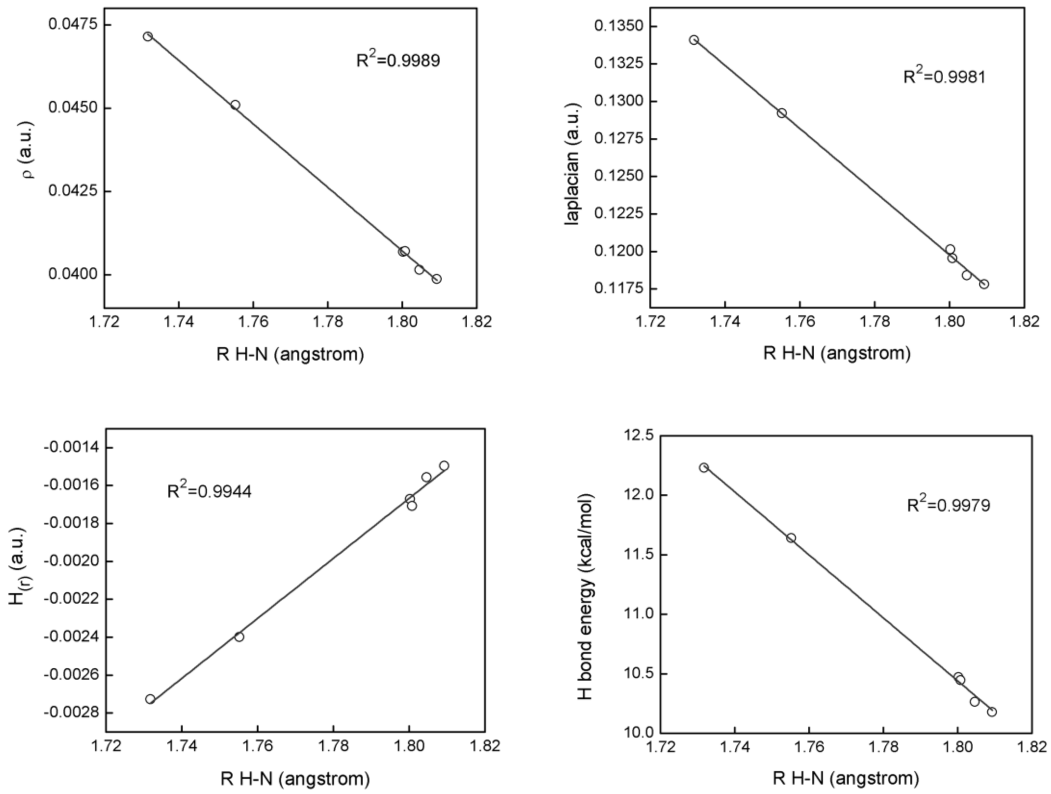

Figure S8. Correlation between H-N bond length and ρ(r), ∇ 2ρ(r), H(r) and hydrogen bond energy estimated via potential energy density V(r) BCP.

6. *Energy levels*

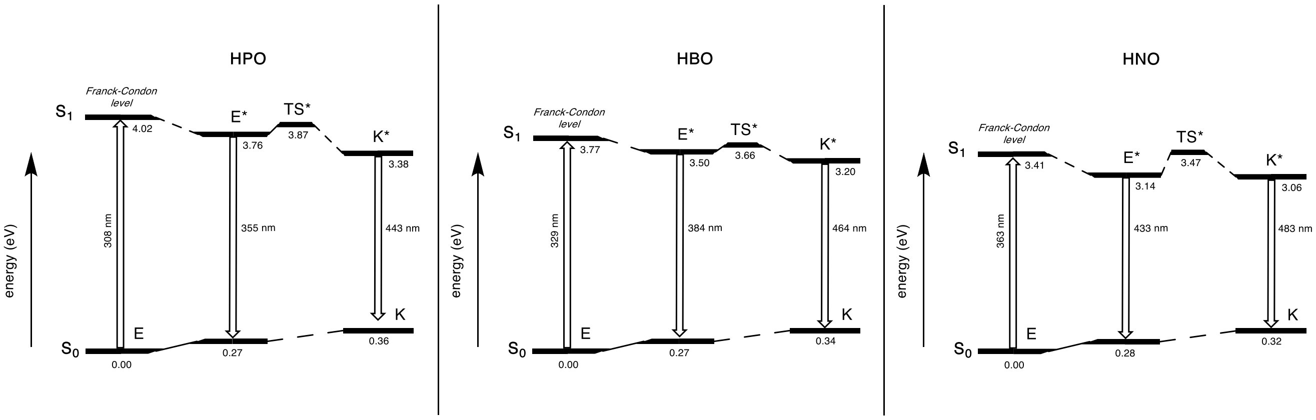

Figure S9. Jablonski diagram (in eV) related to the ESIPT process of **HPO**, **HBO** and **HNO**, calculated at B3LYP/6-31+G(d)//PCM in acetonitrile.

7. *Relevant information about stationary points*

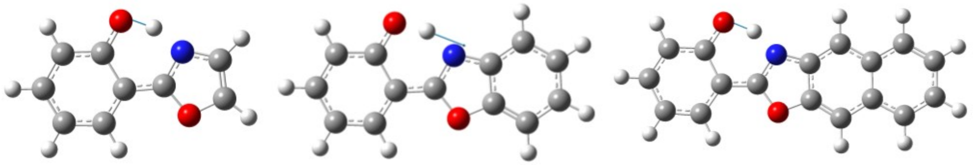

Figure S10. Structures of S_1 transition state for **HPO, HBO** and **HNO** showing the displacement vectors over the imaginary frequency mode.

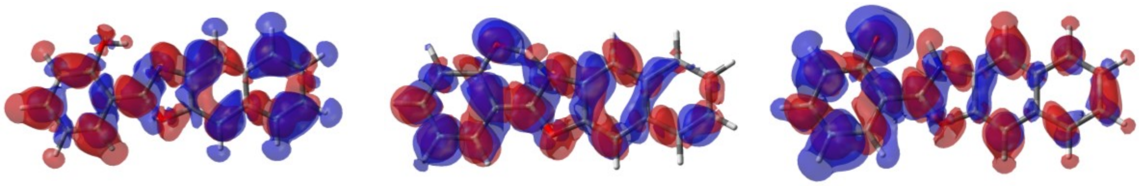

Figure S11. Density difference plots ($\Delta\rho = \rho S_1 - \rho S_0$, isovalue=0.0004) for the E* (left), TS* (middle) and K* (right) stationary points on the S_1 PES of **HNO.** The blue/red zones indicate a decrease/increase of electron density upon excitation, respectively.

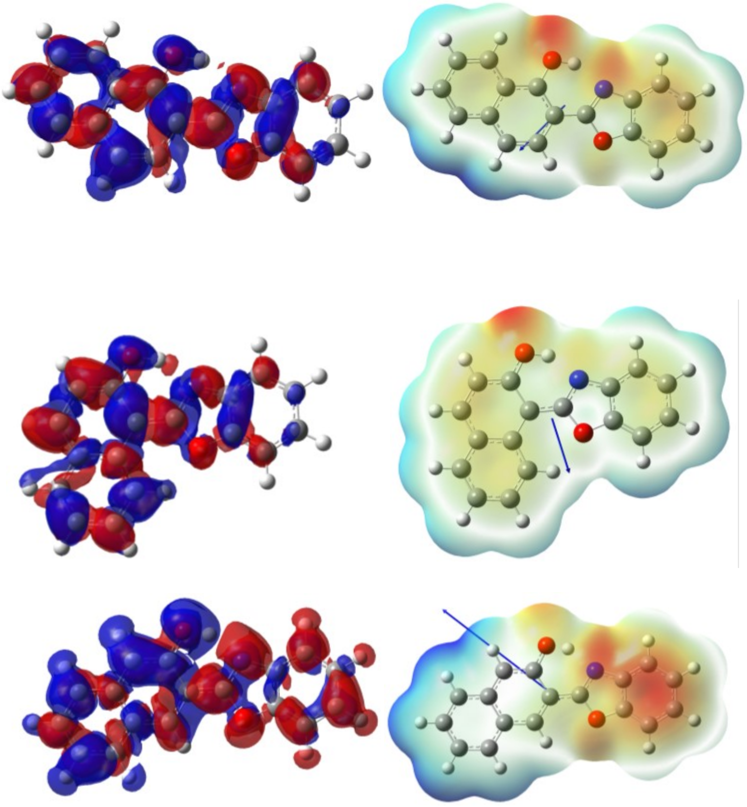

Figure S12. (Left) Density difference plots ($\Delta\rho = \rho S_1 - \rho S_0$, isovalue=0.0004) for the *enol* form of **1H2NBO** (top), **2H1NBO** (middle) and **2H3NBO** (bottom). The blue/red zones indicate a decrease/increase of electron density upon excitation, respectively. (Right) electrostatic potential maps and dipole moment vectors for the three compounds. It is apparent that the ICT character is larger for **2H3NBO** than for its isomers.

8. Optimised coordinates for all compounds

HPO (S_0), *enol* tautomer

C	3.1207120000	0.5437080000	-0.0000780000
C	3.1406930000	-0.8119930000	0.0000240000
O	1.8366470000	-1.2535610000	0.0000720000
N	1.7980640000	0.9691970000	-0.0000950000
C	1.0763120000	-0.1283050000	-0.0000040000
C	-0.3729240000	-0.2200590000	0.0000200000
C	-1.0319890000	-1.4654150000	0.0001220000
C	-1.1458050000	0.9699020000	-0.0000590000
C	-2.4203710000	-1.5389190000	0.0001450000
H	-0.4376110000	-2.3739390000	0.0001830000
C	-2.5449130000	0.8863560000	-0.0000350000
C	-3.1749180000	-0.3555260000	0.0000660000
H	-2.9138030000	-2.5060960000	0.0002240000
H	-3.1190660000	1.8083260000	-0.0000970000
H	-4.2605830000	-0.4028790000	0.0000830000
O	-0.5858580000	2.2067050000	-0.0001590000
H	0.4018020000	2.1164050000	-0.0001660000
H	3.9473010000	1.2390800000	-0.0001390000
H	3.9084120000	-1.5689170000	0.0000720000

HPO (S_1), *enol* tautomer

C	3.0931280000	0.5784970000	-0.0000810000
C	3.1418370000	-0.8053470000	0.0000250000
O	1.8591370000	-1.2874470000	0.0000750000
N	1.8063880000	0.9972030000	-0.0000990000
C	1.0539990000	-0.1446030000	-0.0000040000
C	-0.3371110000	-0.2483390000	0.0000210000
C	-1.0387600000	-1.4997380000	0.0001240000
C	-1.1544110000	0.9791500000	-0.0000590000
C	-2.4272480000	-1.5247730000	0.0001440000
H	-0.4688610000	-2.4220980000	0.0001860000
C	-2.5495950000	0.9144220000	-0.0000360000
C	-3.1958400000	-0.3308100000	0.0000650000
H	-2.9392580000	-2.4834430000	0.0002220000
H	-3.1078360000	1.8458000000	-0.0000980000
H	-4.2802320000	-0.3819060000	0.0000830000
O	-0.5677900000	2.1803660000	-0.0001560000
H	0.4328310000	2.0701700000	-0.0001630000
H	3.9289430000	1.2655770000	-0.0001420000
H	3.9429370000	-1.5286290000	0.0000710000

HPO (S_0), *keto* tautomer

C	-3.1155610000	0.5421110000	0.0000490000

C	-3.1328080000	-0.8054370000	0.0000110000
O	-1.8236440000	-1.2680450000	-0.0000240000
N	-1.7751980000	0.9076510000	0.0000240000
C	-1.0166310000	-0.1931280000	0.0000100000
C	0.4001770000	-0.2386260000	0.0000150000
C	1.0987970000	-1.4736930000	-0.0001330000
C	1.1047630000	1.0345640000	0.0000590000
C	2.4785290000	-1.4997280000	-0.0001510000
H	0.5319650000	-2.4011530000	-0.0001840000
C	2.5390800000	0.9439260000	0.0000420000
C	3.1916050000	-0.2727010000	-0.0000830000
H	3.0132530000	-2.4446750000	-0.0002430000
H	3.0969550000	1.8772200000	0.0001280000
H	4.2796430000	-0.2928250000	-0.0001190000
O	0.4887220000	2.1627470000	0.0001650000
H	-1.2783890000	1.8157670000	0.0001310000
H	-3.9170060000	1.2635480000	0.0000840000
H	-3.9083660000	-1.5527980000	-0.0000070000

HPO (S_1), *keto* tautomer

C	3.1755430000	0.5210140000	0.0005140000
C	3.1481070000	-0.8330520000	-0.0004260000
O	1.8278990000	-1.2623120000	-0.0007670000
N	1.8673800000	0.9454710000	0.0008690000
C	1.0377950000	-0.1393910000	-0.0002050000
C	-0.3868750000	-0.2160110000	-0.0002420000
C	-1.0583930000	-1.4380440000	0.0000110000
C	-1.1748980000	1.0291710000	-0.0007290000
C	-2.4881210000	-1.4977680000	0.0000590000
H	-0.4941730000	-2.3656650000	0.0001960000
C	-2.5950580000	0.9187450000	0.0002270000
C	-3.2493290000	-0.3352350000	0.0003700000
H	-2.9676730000	-2.4723740000	0.0001950000
H	-3.1628560000	1.8454850000	0.0005380000
H	-4.3342770000	-0.3843150000	0.0007860000
O	-0.5736720000	2.1627850000	0.0000020000
H	1.4832690000	1.8877380000	0.0006270000
H	4.0089070000	1.2064980000	0.0010390000
H	3.9087000000	-1.5960210000	-0.0008140000

HPO (S_1), TS

-1264.5 cm^{-1}

C	-3.0567700000	0.6067140000	0.0003850000
C	-3.1705260000	-0.7585850000	0.0002180000
O	-1.8996070000	-1.3157150000	-0.0000280000
N	-1.7332260000	0.9353500000	-0.0001780000
C	-1.0411050000	-0.2368740000	-0.0002200000
C	0.3646780000	-0.3167460000	-0.0003250000

C	1.1270600000	-1.4999120000	-0.0004110000
C	1.0947000000	0.9724900000	0.0000190000
C	2.5386700000	-1.4397760000	-0.0001950000
H	0.6277780000	-2.4632710000	-0.0005970000
C	2.5074900000	0.9881890000	0.0003280000
C	3.2331790000	-0.2142060000	0.0001930000
H	3.0982870000	-2.3711000000	-0.0002570000
H	3.0038470000	1.9541250000	0.0006350000
H	4.3182570000	-0.1998750000	0.0004060000
O	0.4114960000	2.0915180000	0.0001180000
H	-0.7495640000	1.8142570000	-0.0006010000
H	-3.8428670000	1.3483690000	0.0006370000
H	-4.0025370000	-1.4441450000	0.0003530000

HPO K^*_{90} (S_1)

C	3.1306990000	0.1341760000	0.5566760000
C	3.0347490000	-0.1458710000	-0.7542850000
O	1.7499470000	-0.5163750000	-1.0789470000
N	1.8838600000	-0.1125580000	1.1453250000
C	1.0375270000	-0.5345580000	0.1135580000
C	-0.4529480000	-0.3641820000	0.0704210000
C	-1.2902850000	-1.4506440000	0.1433750000
C	-0.9926110000	0.9640410000	-0.0585880000
C	-2.6983080000	-1.2735400000	0.0810370000
H	-0.8751610000	-2.4512430000	0.2418610000
C	-2.4305530000	1.1243050000	-0.1207600000
C	-3.2532350000	0.0133400000	-0.0514930000
H	-3.3501580000	-2.1407330000	0.1334060000
H	-2.8306140000	2.1294660000	-0.2211670000
H	-4.3338820000	0.1243370000	-0.0993230000
O	-0.1972170000	1.9527860000	-0.0943400000
H	1.5332770000	0.5234120000	1.8559880000
H	3.9831690000	0.4549370000	1.1356860000
H	3.7543130000	-0.1459640000	-1.5570810000

HBO (S_0), *enol* tautomer

C	4.3158560000	-0.7382090000	0.0000130000
C	4.2956690000	0.6708030000	-0.0000760000
H	3.1388780000	-2.5746820000	0.0001360000
C	3.1339300000	-1.4897470000	0.0000680000
C	3.0960490000	1.3879400000	-0.0001130000
C	1.9106180000	0.6462580000	-0.0000590000
C	1.9586450000	-0.7545370000	0.0000300000
H	3.0813840000	2.4734810000	-0.0001830000
O	0.6592230000	-1.2169430000	0.0000700000
N	0.5706680000	1.0331150000	-0.0000770000
C	-0.1130860000	-0.0848900000	0.0000070000
C	-1.5569080000	-0.2107480000	0.0000290000

C	-2.1833890000	-1.4741750000	0.0001310000
C	-2.3601440000	0.9604260000	-0.0000510000
C	-3.5682020000	-1.5832800000	0.0001570000
H	-1.5647680000	-2.3660750000	0.0001930000
C	-3.7567860000	0.8393100000	-0.0000250000
C	-4.3526590000	-0.4186110000	0.0000780000
H	-4.0373640000	-2.5623410000	0.0002380000
H	-4.3552280000	1.7455360000	-0.0000880000
H	-5.4367000000	-0.4944140000	0.0000960000
O	-1.8344330000	2.2097060000	-0.0001600000
H	-0.8443580000	2.1460400000	-0.0001730000
H	5.2374510000	1.2122940000	-0.0001180000
H	5.2701450000	-1.2569970000	0.0000380000

HBO (S_1), *enol* tautomer

C	4.3185470000	-0.7250510000	0.0000180000
C	4.2874540000	0.6919560000	-0.0000690000
H	3.1508640000	-2.5759490000	0.0001360000
C	3.1336020000	-1.4911700000	0.0000700000
C	3.0938730000	1.4043990000	-0.0001080000
C	1.8875660000	0.6551940000	-0.0000570000
C	1.9548620000	-0.7773090000	0.0000300000
H	3.0717450000	2.4894090000	-0.0001730000
O	0.6757940000	-1.2615250000	0.0000650000
N	0.5943060000	1.0367530000	-0.0000780000
C	-0.1371540000	-0.1204910000	-0.0000040000
C	-1.5267150000	-0.2388760000	0.0000180000
C	-2.2056250000	-1.5014720000	0.0001210000
C	-2.3590180000	0.9723000000	-0.0000520000
C	-3.5904210000	-1.5570950000	0.0001580000
H	-1.6159010000	-2.4116050000	0.0001760000
C	-3.7511390000	0.8777800000	-0.0000100000
C	-4.3750010000	-0.3760230000	0.0000950000
H	-4.0844190000	-2.5250310000	0.0002390000
H	-4.3280310000	1.7978810000	-0.0000650000
H	-5.4587880000	-0.4426250000	0.0001250000
O	-1.7992920000	2.1899960000	-0.0001730000
H	-0.7998760000	2.0999710000	-0.0002300000
H	5.2297690000	1.2328110000	-0.0001050000
H	5.2774990000	-1.2347490000	0.0000460000

HBO (S_0), *keto* tautomer

C	-4.3069440000	-0.7366980000	-0.0001390000
C	-4.2875450000	0.6694390000	0.0000690000
H	-3.1238330000	-2.5719990000	-0.0002120000
C	-3.1230430000	-1.4874780000	-0.0000520000
C	-3.0883330000	1.3912160000	0.0002910000
C	-1.9141370000	0.6424270000	0.0001710000

C	-1.9501110000	-0.7544590000	0.0000100000
H	-3.0727280000	2.4757980000	0.0003860000
O	-0.6448130000	-1.2317060000	-0.0000380000
N	-0.5611520000	0.9721540000	0.0001040000
C	0.1690630000	-0.1518770000	0.0000580000
C	1.5775930000	-0.2277130000	0.0000190000
C	2.2458640000	-1.4836410000	-0.0001190000
C	2.3198410000	1.0278310000	0.0000740000
C	3.6216290000	-1.5451190000	-0.0000990000
H	1.6542690000	-2.3951750000	-0.0001680000
C	3.7527090000	0.8970290000	-0.0000240000
C	4.3693120000	-0.3358620000	-0.0001720000
H	4.1320620000	-2.5033100000	-0.0001260000
H	4.3355040000	1.8147010000	0.0000460000
H	5.4562200000	-0.3872320000	-0.0001400000
O	1.7394700000	2.1706400000	-0.0000950000
H	-0.0378560000	1.8644850000	0.0002800000
H	-5.2280930000	1.2120040000	0.0000470000
H	-5.2601280000	-1.2563910000	-0.0002950000

HBO (S_1), *keto* tautomer

C	-4.3288860000	-0.7699590000	0.0010860000
C	-4.3363720000	0.6345650000	0.0017920000
H	-3.1001310000	-2.5817250000	-0.0008200000
C	-3.1219060000	-1.4972660000	-0.0001950000
C	-3.1526190000	1.3854510000	0.0012920000
C	-1.9534320000	0.6630540000	0.0000990000
C	-1.9623530000	-0.7454660000	-0.0005780000
H	-3.1623940000	2.4701180000	0.0018090000
O	-0.6591510000	-1.2002580000	-0.0021110000
N	-0.6268530000	1.0212040000	-0.0009800000
C	0.1574320000	-0.0961190000	-0.0026740000
C	1.5798430000	-0.2088370000	-0.0009440000
C	2.2161220000	-1.4480780000	-0.0002910000
C	2.3992240000	1.0168870000	-0.0003380000
C	3.6399820000	-1.5495080000	0.0012620000
H	1.6244220000	-2.3584680000	-0.0010840000
C	3.8183870000	0.8624720000	0.0013620000
C	4.4330520000	-0.4056240000	0.0021510000
H	4.0931880000	-2.5363260000	0.0016700000
H	4.4131240000	1.7719660000	0.0020950000
H	5.5161360000	-0.4860760000	0.0033710000
O	1.8361920000	2.1670700000	-0.0013710000
H	-0.1843890000	1.9404610000	-0.0006870000
H	-5.2884160000	1.1577950000	0.0027550000
H	-5.2707520000	-1.3101010000	0.0014700000

HBO (S_1), TS
-1346.5 cm^{-1}

C	4.3339950000	-0.6673360000	0.0001440000
C	4.2542450000	0.7403780000	-0.0000910000
H	3.2218800000	-2.5524340000	0.0004150000
C	3.1733870000	-1.4685130000	0.0002210000
C	3.0314280000	1.4157100000	-0.0002790000
C	1.8625070000	0.6275870000	-0.0002250000
C	1.9701930000	-0.7920640000	0.0000360000
H	2.9764210000	2.4994410000	-0.0004460000
O	0.7017580000	-1.3299060000	0.0000530000
N	0.5373590000	0.9345210000	-0.0004410000
C	-0.1509290000	-0.2406220000	-0.0001490000
C	-1.5557600000	-0.3163460000	-0.0000140000
C	-2.3135910000	-1.5015420000	0.0000120000
C	-2.2817030000	0.9746700000	0.0001060000
C	-3.7204400000	-1.4475460000	0.0001200000
H	-1.8080990000	-2.4618280000	-0.0000770000
C	-3.6948200000	0.9819760000	0.0000780000
C	-4.4134840000	-0.2194140000	0.0001390000
H	-4.2802870000	-2.3784900000	0.0001310000
H	-4.1965560000	1.9451610000	0.0001100000
H	-5.4988490000	-0.2065980000	0.0001810000
O	-1.6018310000	2.0938830000	0.0002340000
H	-0.4287110000	1.8092310000	-0.0002730000
H	5.1749430000	1.3175560000	-0.0001190000
H	5.3081640000	-1.1471350000	0.0002850000

HBO K^*_{90} (S_1)

C	4.1882390000	0.2706800000	-0.7900870000
C	4.2618530000	0.2094810000	0.6074000000
H	2.8981880000	0.1046770000	-2.5458470000
C	2.9695510000	0.0660270000	-1.4637390000
C	3.1279740000	-0.0523100000	1.3940330000
C	1.9204690000	-0.2482220000	0.7242650000
C	1.8608390000	-0.1892870000	-0.6754500000
H	3.1888530000	-0.0988170000	2.4768100000
O	0.5715970000	-0.4287920000	-1.0967630000
N	0.6283110000	-0.5022230000	1.1542620000
C	-0.1839340000	-0.7580570000	0.0395520000
C	-1.6293840000	-0.4102070000	0.0051180000
C	-2.5799590000	-1.4083380000	-0.0845670000
C	-2.0589420000	0.9849450000	0.0624290000
C	-3.9592350000	-1.1038840000	-0.1087280000
H	-2.2598500000	-2.4457780000	-0.1331570000
C	-3.4843410000	1.2590580000	0.0391130000
C	-4.4019310000	0.2335020000	-0.0436510000
H	-4.6829670000	-1.9096860000	-0.1766520000
H	-3.7948870000	2.2988880000	0.0900380000
H	-5.4667220000	0.4474080000	-0.0607820000
O	-1.2161020000	1.9253770000	0.1243710000

H	0.3856450000	-0.8168840000	2.0835250000
H	5.2176730000	0.3673700000	1.0989730000
H	5.0847260000	0.4753720000	-1.3677330000

HNO (S_0), *enol* tautomer

C	-5.5685237194	-0.7024964338	-0.0000727099
C	-4.3892456484	-1.4156569367	-0.0000025150
C	-3.1281242101	-0.7529141363	-0.0000211004
C	-3.1045937952	0.6933514367	-0.0001158594
C	-4.3473681728	1.3937903343	-0.0001858579
C	-5.5473755227	0.7175605532	-0.0001651989
H	-1.9142606937	-2.5779412490	0.0001247579
H	-6.5205146285	-1.2266154753	-0.0000575201
H	-4.4050410801	-2.5030036814	0.0000674151
C	-1.9096937991	-1.4924076031	0.0000523833
C	-1.8675140628	1.3937048246	-0.0001371307
H	-4.3294447133	2.4811634161	-0.0002558271
H	-6.4833256418	1.2697960551	-0.0002198680
C	-0.7034048882	0.6541703889	-0.0000663503
C	-0.7534020112	-0.7628040350	0.0000270027
H	-1.8492661753	2.4797578368	-0.0002061717
O	0.5499442281	-1.2214459485	0.0000886198
N	0.6402416775	1.0340464922	-0.0000635720
C	1.3197586969	-0.0845418768	0.0000137254
C	2.7619409799	-0.2112555776	0.0000691330
C	3.3873222078	-1.4757746585	0.0002331122
C	3.5656774170	0.9602842271	-0.0000204649
C	4.7716453025	-1.5853841285	0.0003156804
H	2.7676658147	-2.3668285577	0.0003041176
C	4.9623119148	0.8379289949	0.0000674159
C	5.5565439941	-0.4205988210	0.0002336988
H	5.5615424962	1.7435659596	-0.0000071120
H	6.6405267275	-0.4971911406	0.0002963120
O	3.0404872573	2.2085342606	-0.0002228662
H	2.0503783646	2.1448774123	-0.0003234309
H	5.2408677121	-2.5643518354	0.0004465176

HBO (S_1), *enol* tautomer

C	-5.5909410000	-0.6881030000	-0.0000830000
C	-4.3759370000	-1.4073100000	0.0000120000
C	-3.1407880000	-0.7531910000	-0.0000130000
C	-3.1132350000	0.6947090000	-0.0001410000
C	-4.3609260000	1.3924820000	-0.0002350000
C	-5.5761990000	0.7103530000	-0.0002060000
H	-1.9110150000	-2.5821080000	0.0001800000
H	-6.5327740000	-1.2282000000	-0.0000600000
H	-4.3995540000	-2.4938450000	0.0001060000
C	-1.9002770000	-1.4967190000	0.0000850000

C	-1.8904240000	1.3905320000	-0.0001710000
H	-4.3472770000	2.4792720000	-0.0003300000
H	-6.5082800000	1.2673830000	-0.0002790000
C	-0.6792680000	0.6453690000	-0.0000740000
C	-0.7409120000	-0.7788000000	0.0000520000
H	-1.8679020000	2.4762470000	-0.0002640000
O	0.5467940000	-1.2468770000	0.0001270000
N	0.6079600000	1.0397430000	-0.0000770000
C	1.3506520000	-0.1125430000	0.0000450000
C	2.7483600000	-0.2218710000	0.0000910000
C	3.4185170000	-1.4893680000	0.0002250000
C	3.5717820000	0.9689890000	0.0000010000
C	4.8012930000	-1.5661190000	0.0002670000
H	2.8191660000	-2.3941700000	0.0002960000
C	4.9590590000	0.8630120000	0.0000450000
C	5.5840340000	-0.3932960000	0.0001770000
H	5.5444000000	1.7785870000	-0.0000260000
H	6.6683460000	-0.4566160000	0.0002090000
O	3.0246050000	2.2134890000	-0.0001260000
H	2.0351110000	2.1335170000	-0.0001400000
H	5.2841260000	-2.5399140000	0.0003700000

HNO (S_0), *keto* tautomer

C	5.5602760000	-0.7059750000	0.0001780000
C	4.3791730000	-1.4171950000	0.0001280000
C	3.1217530000	-0.7503740000	0.0000320000
C	3.1011030000	0.6939500000	-0.0000050000
C	4.3425610000	1.3927400000	0.0000460000
C	5.5414070000	0.7126980000	0.0001370000
H	1.8998230000	-2.5731940000	-0.0000130000
H	6.5110780000	-1.2318900000	0.0002520000
H	4.3919450000	-2.5043260000	0.0001610000
C	1.9002850000	-1.4880920000	-0.0000300000
C	1.8637990000	1.3998660000	-0.0000910000
H	4.3273230000	2.4798790000	0.0000200000
H	6.4781280000	1.2633740000	0.0001780000
C	0.7101470000	0.6543450000	-0.0001110000
C	0.7468460000	-0.7596110000	-0.0000900000
H	1.8458890000	2.4850180000	-0.0001360000
O	-0.5622680000	-1.2319330000	-0.0001360000
N	-0.6455160000	0.9762890000	-0.0002790000
C	-1.3743850000	-0.1489930000	-0.0002030000
C	-2.7796560000	-0.2269090000	-0.0001370000
C	-3.4446630000	-1.4863360000	-0.0001440000
C	-3.5276140000	1.0273950000	0.0000100000
C	-4.8189550000	-1.5511990000	0.0000450000
H	-2.8501310000	-2.3958150000	-0.0003200000
C	-4.9607250000	0.8912370000	0.0003190000
C	-5.5714900000	-0.3433910000	0.0003100000

H	-5.3268420000	-2.5107140000	-0.0000060000
H	-5.5467020000	1.8067060000	0.0005300000
H	-6.6581360000	-0.3992040000	0.0005070000
O	-2.9534240000	2.1710690000	-0.0000490000
H	-1.1674090000	1.8681210000	-0.0000990000

HNO (S_1), *keto* tautomer

C	-5.5859960000	-0.7365140000	-0.0003620000
C	-4.3861130000	-1.4306940000	-0.0004380000
C	-3.1414080000	-0.7519940000	-0.0002680000
C	-3.1371720000	0.6979700000	-0.0000080000
C	-4.3908600000	1.3748480000	0.0000630000
C	-5.5853730000	0.6775660000	-0.0001090000
H	-1.8916280000	-2.5613580000	-0.0005460000
H	-6.5277190000	-1.2788780000	-0.0004970000
H	-4.3844680000	-2.5183750000	-0.0006320000
C	-1.9046860000	-1.4764620000	-0.0003510000
C	-1.9108750000	1.4203200000	0.0001680000
H	-4.3922390000	2.4624560000	0.0002550000
H	-6.5282250000	1.2185000000	-0.0000490000
C	-0.7343250000	0.6847800000	0.0000870000
C	-0.7552920000	-0.7351210000	-0.0001690000
H	-1.9062140000	2.5054610000	0.0003560000
O	0.5487190000	-1.1910160000	-0.0002090000
N	0.5995220000	1.0194940000	0.0001980000
C	1.3668930000	-0.0987310000	0.0000070000
C	2.7937920000	-0.2150060000	0.0000840000
C	3.4220630000	-1.4553400000	0.0000120000
C	3.6112600000	1.0093410000	0.0002320000
C	4.8424360000	-1.5618410000	0.0000790000
H	2.8258310000	-2.3626950000	-0.0000980000
C	5.0339480000	0.8488940000	0.0003330000
C	5.6390930000	-0.4171430000	0.0002460000
H	5.6317700000	1.7560670000	0.0004660000
H	6.7217450000	-0.5028230000	0.0003110000
O	3.0566390000	2.1598690000	0.0003450000
H	1.0532380000	1.9341250000	0.0003190000
H	5.2940720000	-2.5490020000	0.0000090000

HNO (S_1), TS
-1581.4 cm^{-1}

C	-5.5816090000	-0.6186570000	0.0001000000
C	-4.4044790000	-1.3687710000	0.0001370000
C	-3.1403570000	-0.7501730000	0.0000130000
C	-3.0716770000	0.7012350000	-0.0001930000
C	-4.3010090000	1.4359080000	-0.0002150000
C	-5.5243800000	0.7915310000	-0.0000760000
H	-1.9711900000	-2.6158130000	0.0002400000

H	-6.5434720000	-1.1238490000	0.0002120000
H	-4.4560300000	-2.4549050000	0.0002780000
C	-1.9290840000	-1.5314140000	0.0000780000
C	-1.8267890000	1.3636920000	-0.0003520000
H	-4.2529640000	2.5221950000	-0.0003490000
H	-6.4435260000	1.3710260000	-0.0000980000
C	-0.6584260000	0.5796580000	-0.0003170000
C	-0.7510680000	-0.8491330000	-0.0000680000
H	-1.7718630000	2.4477950000	-0.0004710000
O	0.5306560000	-1.3642670000	-0.0000720000
N	0.6577260000	0.8919730000	-0.0005420000
C	1.3671140000	-0.2729100000	-0.0003340000
C	2.7719740000	-0.3088000000	0.0001520000
C	3.5506720000	-1.4855750000	0.0002640000
C	3.4585420000	0.9983340000	0.0001770000
C	4.9471850000	-1.4099400000	0.0003860000
H	3.0560040000	-2.4519610000	0.0001870000
C	4.8721830000	1.0233440000	0.0001370000
C	5.6090470000	-0.1620560000	0.0002990000
H	5.3613950000	1.9932300000	0.0001240000
H	6.6945040000	-0.1256570000	0.0003390000
O	2.7588110000	2.1053790000	0.0002990000
H	1.5514140000	1.7749740000	-0.0000650000
H	5.5288920000	-2.3273710000	0.0004570000

HNO K^*_{90} (S_1)

C	-5.5274080000	-0.7969840000	0.2628370000
C	-4.3040970000	-1.4471070000	0.2338820000
C	-3.0935070000	-0.7354520000	0.0485370000
C	-3.1495570000	0.6987270000	-0.1133940000
C	-4.4204230000	1.3307130000	-0.0783680000
C	-5.5839490000	0.6032860000	0.1052420000
H	-1.7710690000	-2.4886280000	0.1527840000
H	-6.4421140000	-1.3655360000	0.4081840000
H	-4.2573940000	-2.5271560000	0.3559840000
C	-1.8301210000	-1.4131750000	0.0234610000
C	-1.9509910000	1.4520310000	-0.3065010000
H	-4.4665510000	2.4110910000	-0.1986160000
H	-6.5434960000	1.1135280000	0.1292470000
C	-0.7521610000	0.7625080000	-0.3231940000
C	-0.7108450000	-0.6460840000	-0.1557170000
H	-1.9974980000	2.5300170000	-0.4284700000
O	0.5981090000	-1.0668140000	-0.2282310000
N	0.5644810000	1.1360240000	-0.5117110000
C	1.3769380000	0.0290420000	-0.4828230000
C	2.8593800000	-0.0177770000	-0.3093400000
C	3.6939230000	-0.2389470000	-1.3782870000
C	3.4079670000	0.1668440000	1.0152320000
C	5.1012590000	-0.3003440000	-1.1943640000

H	3.2771040000	-0.3785160000	-2.3726580000
C	4.8442920000	0.0917520000	1.1779290000
C	5.6610470000	-0.1366930000	0.0828280000
H	5.2470860000	0.2212560000	2.1781070000
H	6.7397190000	-0.1918550000	0.2050730000
O	2.6365320000	0.4053900000	1.9892260000
H	0.9107510000	2.0838500000	-0.5336390000
H	5.7444800000	-0.4828590000	-2.0497290000

1H2NBO (S_0), *enol* tautomer

O	1.9419620000	-0.8478170000	0.0000000000
C	1.3882840000	0.3831020000	0.0000000000
C	0.0000000000	0.5919440000	0.0000000000
C	-0.5131370000	1.9250990000	0.0000000000
C	0.3282380000	3.0068720000	0.0000000000
C	2.2856910000	1.4984460000	0.0000000000
C	1.7443050000	2.8236430000	0.0000000000
C	-0.8883370000	-0.5490910000	0.0000000000
C	3.6946920000	1.3172150000	0.0000000000
N	-0.5355050000	-1.8139140000	0.0000000000
C	-1.7326210000	-2.5291590000	0.0000000000
O	-2.2437610000	-0.3422460000	0.0000000000
H	-1.5890310000	2.0669400000	0.0000000000
C	-2.7905790000	-1.6086750000	0.0000000000
C	-1.9997740000	-3.9021390000	0.0000000000
C	-3.3426960000	-4.2898030000	0.0000000000
H	-3.5899080000	-5.3476890000	0.0000000000
C	-4.3872980000	-3.3432790000	0.0000000000
C	-4.1300110000	-1.9663840000	0.0000000000
H	-1.1963360000	-4.6325520000	0.0000000000
H	-5.4173920000	-3.6877910000	0.0000000000
H	-4.9265240000	-1.2293840000	0.0000000000
C	2.6434790000	3.9233620000	0.0000000000
H	-0.0739460000	4.0162650000	0.0000000000
C	4.5407640000	2.4091780000	0.0000000000
C	4.0104740000	3.7229020000	0.0000000000
H	2.2351810000	4.9312520000	0.0000000000
H	4.0967610000	0.3095950000	0.0000000000
H	5.6173830000	2.2621190000	0.0000000000
H	4.6844760000	4.5755270000	0.0000000000
H	1.2134090000	-1.5257740000	0.0000000000

1H2NBO (S_1), *enol* tautomer

O	1.8212120000	-1.0077040000	0.0000000000
C	1.4275720000	0.2725440000	0.0000000000
C	0.0000000000	0.6054120000	0.0000000000
C	-0.3481310000	1.9810920000	0.0000000000
C	0.6141420000	2.9722870000	0.0000000000

C	2.4139760000	1.2795410000	0.0000000000
C	2.0141140000	2.6664440000	0.0000000000
C	-0.9583840000	-0.4231200000	0.0000000000
C	3.8086130000	0.9774460000	0.0000000000
N	-0.7120950000	-1.7526680000	0.0000000000
C	-1.9332570000	-2.3577620000	0.0000000000
O	-2.3208000000	-0.1298020000	0.0000000000
H	-1.3987420000	2.2526160000	0.0000000000
C	-2.9407350000	-1.3550200000	0.0000000000
C	-2.3116940000	-3.7175820000	0.0000000000
C	-3.6778250000	-4.0038560000	0.0000000000
H	-4.0014880000	-5.0413310000	0.0000000000
C	-4.6547930000	-2.9859380000	0.0000000000
C	-4.2955960000	-1.6230120000	0.0000000000
H	-1.5638710000	-4.5046510000	0.0000000000
H	-5.7069140000	-3.2555750000	0.0000000000
H	-5.0391980000	-0.8326110000	0.0000000000
C	2.9950430000	3.6677640000	0.0000000000
H	0.3066910000	4.0145450000	0.0000000000
C	4.7564410000	1.9981750000	0.0000000000
C	4.3640580000	3.3478080000	0.0000000000
H	2.6823420000	4.7093060000	0.0000000000
H	4.1270690000	-0.0597860000	0.0000000000
H	5.8127620000	1.7422540000	0.0000000000
H	5.1088920000	4.1377230000	0.0000000000
H	1.0125570000	-1.6071000000	0.0000000000

2H1NBO (S_0), *enol* tautomer

C	2.7607690000	0.5535820000	0.0000070000
C	2.4559720000	-0.8122350000	0.0000130000
C	3.4070980000	-1.8208520000	0.0000310000
C	4.7402050000	-1.3918030000	0.0000300000
C	5.0750110000	-0.0224050000	0.0000270000
C	4.0951240000	0.9737850000	0.0000210000
C	0.6048400000	0.3547330000	-0.0001100000
H	3.1379690000	-2.8719340000	0.0000430000
H	5.5334760000	-2.1335790000	0.0000450000
H	6.1229150000	0.2638580000	0.0000460000
H	4.3544210000	2.0280580000	0.0000390000
C	-0.8208450000	0.6411950000	-0.0000860000
C	-1.8685400000	-0.3671940000	-0.0000490000
C	-1.1896670000	2.0062070000	-0.0000310000
C	-1.6376910000	-1.7728170000	-0.0000880000
C	-3.2392800000	0.0632440000	0.0000350000
C	-2.5533400000	2.3974450000	0.0000630000
C	-2.6852720000	-2.6781730000	-0.0000550000
H	-0.6288430000	-2.1560360000	-0.0001490000
C	-4.2912520000	-0.8898950000	0.0000690000
C	-3.5441840000	1.4547580000	0.0000920000

H	-2.7756830000	3.4600160000	0.0001040000
C	-4.0278420000	-2.2440890000	0.0000240000
H	-2.4627080000	-3.7422040000	-0.0000910000
H	-5.3161810000	-0.5261510000	0.0001330000
H	-4.5871310000	1.7612700000	0.0001640000
H	-4.8392780000	-2.9664380000	0.0000520000
O	1.0828360000	-0.9314990000	0.0000170000
N	1.5627760000	1.2592060000	0.0000410000
O	-0.3063130000	3.0245700000	-0.0000710000
H	0.6228040000	2.6511980000	-0.0001950000

2H1NBO (S_1), *enol* tautomer

C	2.7461950000	0.5582630000	-0.0000750000
C	2.4437360000	-0.8311020000	0.0000580000
C	3.3964200000	-1.8293990000	0.0000800000
C	4.7363120000	-1.3963210000	-0.0000370000
C	5.0708250000	-0.0218860000	-0.0001710000
C	4.0980880000	0.9740450000	-0.0001930000
C	0.5791840000	0.3388920000	0.0000850000
H	3.1319010000	-2.8816170000	0.0001780000
H	5.5307810000	-2.1366920000	-0.0000260000
H	6.1200670000	0.2601310000	-0.0002600000
H	4.3567620000	2.0281870000	-0.0002970000
C	-0.7967310000	0.6245800000	0.0000890000
C	-1.8737060000	-0.3730970000	0.0000400000
C	-1.1916110000	2.0480670000	0.0000690000
C	-1.6504960000	-1.7662180000	0.0000360000
C	-3.2438770000	0.0802270000	-0.0000340000
C	-2.5266580000	2.4249660000	-0.0000110000
C	-2.7070070000	-2.6952530000	-0.0000220000
H	-0.6412480000	-2.1499650000	0.0000780000
C	-4.2875830000	-0.8763340000	-0.0000920000
C	-3.5445290000	1.4657900000	-0.0000610000
H	-2.7569560000	3.4859410000	-0.0000200000
C	-4.0287130000	-2.2496000000	-0.0000840000
H	-2.4799950000	-3.7572400000	-0.0000210000
H	-5.3148510000	-0.5193610000	-0.0001470000
H	-4.5845310000	1.7805950000	-0.0001210000
H	-4.8530520000	-2.9573710000	-0.0001310000
O	1.0804110000	-0.9616910000	0.0001370000
N	1.5854610000	1.2558850000	-0.0000590000
O	-0.2769020000	3.0329780000	0.0001970000
H	0.6457190000	2.6321910000	0.0004400000

2H1NBO (S_0), *keto* tautomer

C	2.7580540000	0.5439800000	-0.0001200000
C	2.4403170000	-0.8070010000	0.0000920000
C	3.3846900000	-1.8074940000	0.0002290000

C	4.7147770000	-1.3895480000	0.0001420000
C	5.0506450000	-0.0343170000	-0.0000890000
C	4.0784810000	0.9625030000	-0.0002340000
C	0.5469480000	0.3051250000	-0.0000140000
H	3.1075200000	-2.8534870000	0.0003930000
H	5.5016990000	-2.1336720000	0.0002450000
H	6.0961270000	0.2499910000	-0.0001620000
H	4.3398140000	2.0128580000	-0.0004200000
C	-0.8137580000	0.6177120000	0.0000010000
C	-1.8846880000	-0.3714540000	-0.0000380000
C	-1.1099120000	2.0418410000	0.0001410000
C	-1.6838050000	-1.7673890000	-0.0001230000
C	-3.2234600000	0.0890350000	-0.0000380000
C	-2.5105690000	2.4233930000	0.0001400000
C	-2.7493860000	-2.6458610000	-0.0001900000
H	-0.6841000000	-2.1720850000	-0.0001440000
C	-4.2905250000	-0.8227510000	-0.0001060000
C	-3.4916270000	1.5036640000	0.0000380000
H	-2.7150450000	3.4872990000	0.0002610000
C	-4.0667820000	-2.1815310000	-0.0001810000
H	-2.5537650000	-3.7127110000	-0.0002560000
H	-5.3040450000	-0.4336930000	-0.0001010000
H	-4.5310340000	1.8197310000	0.0000610000
H	-4.8962350000	-2.8788490000	-0.0002370000
O	1.0709700000	-0.9383200000	0.0001100000
N	1.5439110000	1.2068740000	-0.0002190000
O	-0.2324730000	2.9390820000	0.0004490000
H	1.2273160000	2.2009770000	-0.0004610000

2H1NBO (S_1), *keto* tautomer

C	2.7618480000	0.5605500000	0.0000310000
C	2.4494000000	-0.8008610000	-0.0000170000
C	3.4007700000	-1.7949800000	-0.0000550000
C	4.7312420000	-1.3679900000	-0.0000410000
C	5.0601720000	-0.0099650000	0.0000140000
C	4.0873900000	0.9853070000	0.0000550000
C	0.5483770000	0.3066050000	0.0000230000
H	3.1296920000	-2.8424970000	-0.0000900000
H	5.5225850000	-2.1074560000	-0.0000690000
H	6.1050930000	0.2772380000	0.0000280000
H	4.3448870000	2.0364620000	0.0001010000
C	-0.8343810000	0.6071860000	0.0000340000
C	-1.8787590000	-0.3629490000	0.0000200000
C	-1.1812810000	2.0679810000	0.0000430000
C	-1.6524380000	-1.7693920000	0.0000010000
C	-3.2552720000	0.0953100000	0.0000290000
C	-2.5536410000	2.4255090000	-0.0000270000
C	-2.6890000000	-2.6732450000	0.0000010000
H	-0.6414060000	-2.1451980000	-0.0000130000

C	-4.2843140000	-0.8607270000	0.0000270000
C	-3.5528010000	1.4793400000	-0.0000030000
H	-2.7709820000	3.4864980000	-0.0000940000
C	-4.0190650000	-2.2143910000	0.0000170000
H	-2.4791420000	-3.7359490000	-0.0000160000
H	-5.3113990000	-0.5105310000	0.0000280000
H	-4.5940670000	1.7827140000	-0.0000310000
H	-4.8372720000	-2.9259400000	0.0000140000
O	1.0887870000	-0.9414250000	-0.0000140000
N	1.5591080000	1.2101260000	0.0000680000
O	-0.2717790000	2.9377550000	-0.0001600000
H	1.2527140000	2.2034170000	0.0001560000

2H3NBO (S_0), *enol* tautomer

O	-2.1496200000	1.6911080000	0.0000000000
C	-0.7914650000	1.7259140000	0.0000000000
C	0.0000000000	0.5229380000	0.0000000000
C	1.3863240000	0.6181340000	0.0000000000
C	2.0441180000	1.8683320000	0.0000000000
C	-0.1645820000	2.9565680000	0.0000000000
C	1.2481510000	3.0644080000	0.0000000000
C	-0.6603500000	-0.7696120000	0.0000000000
C	3.4651580000	1.9749580000	0.0000000000
H	-0.7764590000	3.8546020000	0.0000000000
C	1.9133140000	4.3240290000	0.0000000000
N	-1.9520860000	-0.9845540000	0.0000000000
C	-2.0955200000	-2.3715460000	0.0000000000
O	0.1001480000	-1.9089980000	0.0000000000
H	1.9804120000	-0.2909380000	0.0000000000
C	-0.8147200000	-2.9405800000	0.0000000000
C	-3.2274660000	-3.1926260000	0.0000000000
C	-3.0116940000	-4.5728670000	0.0000000000
H	-3.8656790000	-5.2441080000	0.0000000000
C	-1.7123430000	-5.1194120000	0.0000000000
C	-0.5730170000	-4.3056630000	0.0000000000
H	-4.2283050000	-2.7720630000	0.0000000000
H	-1.5899630000	-6.1985550000	0.0000000000
H	0.4311660000	-4.7163270000	0.0000000000
H	1.3169160000	5.2333150000	0.0000000000
C	3.2906520000	4.3931390000	0.0000000000
C	4.0770280000	3.2093040000	0.0000000000
H	4.0586020000	1.0636320000	0.0000000000
H	3.7832470000	5.3620180000	0.0000000000
H	5.1609700000	3.2831630000	0.0000000000
H	-2.4520490000	0.7477330000	0.0000000000

2H3NBO (S_1), *enol* tautomer

O	-2.2824350000	1.2428860000	0.0000000000

C	-0.9854360000	1.5144940000	0.0000000000
C	0.0000000000	0.4457600000	0.0000000000
C	1.3497150000	0.8376620000	0.0000000000
C	1.7559680000	2.2073870000	0.0000000000
C	-0.6031840000	2.8716300000	0.0000000000
C	0.7591270000	3.2516560000	0.0000000000
C	-0.4628890000	-0.8902980000	0.0000000000
C	3.1057180000	2.5911210000	0.0000000000
H	-1.3898980000	3.6206790000	0.0000000000
C	1.1583270000	4.6090720000	0.0000000000
N	-1.7513060000	-1.2827990000	0.0000000000
C	-1.7161460000	-2.6612270000	0.0000000000
O	0.4319250000	-1.9513530000	0.0000000000
H	2.1212610000	0.0728500000	0.0000000000
C	-0.3670110000	-3.0789970000	0.0000000000
C	-2.7340610000	-3.6283590000	0.0000000000
C	-2.3490940000	-4.9765650000	0.0000000000
H	-3.1183980000	-5.7447940000	0.0000000000
C	-0.9977240000	-5.3633960000	0.0000000000
C	0.0335010000	-4.4023010000	0.0000000000
H	-3.7811700000	-3.3391270000	0.0000000000
H	-0.7396810000	-6.4186770000	0.0000000000
H	1.0818440000	-4.6853300000	0.0000000000
H	0.3920840000	5.3803370000	0.0000000000
C	2.5174300000	4.9653290000	0.0000000000
C	3.4815850000	3.9554190000	0.0000000000
H	3.8781240000	1.8261930000	0.0000000000
H	2.8096040000	6.0104620000	0.0000000000
H	4.5376910000	4.2101830000	0.0000000000
H	-2.4031970000	0.2242300000	0.0000000000

2H3NBO (S_0), *keto* tautomer

O	-2.2821740000	1.2917440000	0.0000000000
C	-1.0213330000	1.5721850000	0.0000000000
C	0.0000000000	0.5131300000	0.0000000000
C	1.3689330000	0.8035210000	0.0000000000
C	1.8283730000	2.1272530000	0.0000000000
C	-0.5225460000	2.8953470000	0.0000000000
C	0.8496830000	3.1939140000	0.0000000000
C	-0.4582310000	-0.8320500000	0.0000000000
C	3.2220890000	2.4484320000	0.0000000000
H	-1.2469160000	3.7069350000	0.0000000000
C	1.3353060000	4.5425780000	0.0000000000
N	-1.7375930000	-1.2069220000	0.0000000000
C	-1.8061930000	-2.5979510000	0.0000000000
O	0.3498110000	-1.9106590000	0.0000000000
H	2.0900690000	-0.0101700000	0.0000000000
C	-0.4763590000	-3.0285700000	0.0000000000
C	-2.8583600000	-3.5113010000	0.0000000000

C	-2.5053180000	-4.8645230000	0.0000000000
H	-3.2911940000	-5.6136790000	0.0000000000
C	-1.1611800000	-5.2817480000	0.0000000000
C	-0.1050520000	-4.3615060000	0.0000000000
H	-3.8938870000	-3.1886530000	0.0000000000
H	-0.9341380000	-6.3433490000	0.0000000000
H	0.9345460000	-4.6701840000	0.0000000000
H	0.6124520000	5.3552790000	0.0000000000
C	2.6831740000	4.8116370000	0.0000000000
C	3.6444210000	3.7555460000	0.0000000000
H	3.9429580000	1.6334520000	0.0000000000
H	3.0273620000	5.8430750000	0.0000000000
H	4.7047320000	3.9925680000	0.0000000000
H	-2.4283740000	-0.4208670000	0.0000000000

2H3NBO (S_1), *keto* tautomer

O	-0.3031250000	2.6649080000	-0.0000200000
C	-1.0482490000	1.6288200000	-0.0000060000
C	-0.4460180000	0.2755960000	0.0000010000
C	-1.2809470000	-0.8303090000	0.0000030000
C	-2.7184130000	-0.7261730000	0.0000020000
C	-2.4644480000	1.7177110000	-0.0000060000
C	-3.3195320000	0.5788160000	-0.0000020000
C	0.9650550000	0.1462000000	0.0000030000
C	-3.5434240000	-1.8555270000	0.0000050000
H	-2.8958410000	2.7156610000	-0.0000130000
C	-4.7249440000	0.6834900000	-0.0000020000
N	1.9267100000	1.1157040000	0.0000180000
C	3.1779430000	0.5312370000	0.0000070000
O	1.5812320000	-1.0809450000	-0.0000030000
H	-0.8480300000	-1.8276190000	0.0000070000
C	2.9464460000	-0.8531310000	-0.0000010000
C	4.4792120000	1.0366300000	0.0000100000
C	5.5205660000	0.0965320000	0.0000020000
H	6.5471640000	0.4515330000	0.0000040000
C	5.2731090000	-1.2843960000	-0.0000070000
C	3.9600940000	-1.7918840000	-0.0000080000
H	4.6746620000	2.1037890000	0.0000180000
H	6.1077370000	-1.9788150000	-0.0000120000
H	3.7527180000	-2.8566840000	-0.0000160000
H	-5.1802950000	1.6709310000	-0.0000050000
C	-5.5358870000	-0.4632000000	0.0000010000
C	-4.9480570000	-1.7285620000	0.0000050000
H	-3.0930780000	-2.8452630000	0.0000080000
H	-6.6172540000	-0.3608470000	0.0000010000
H	-5.5669490000	-2.6212740000	0.0000070000
H	1.6523130000	2.0958520000	0.0000100000

[i] E. Espinosa, E. Molins, C. Lecomte, *Chem. Phys. Lett.* 1998, **285**, 170– 173.

[ii] A. J. Stasyuk, M. K. Cyrański, D. T. Gryko, M. Solà, *J. Chem. Theory Comput.,* 2015, **11**, 1046-1054.
